# Modeling exposure–lag–response associations with distributed lag non-linear models

**DOI:** 10.1002/sim.5963

**Published:** 2013-09-12

**Authors:** Antonio Gasparrini

**Affiliations:** Medical Statistics Department, London School of Hygiene and Tropical MedicineLondon, U.K.

**Keywords:** latency, distributed lag models, exposure–lag–response, delayed effects, splines

## Abstract

In biomedical research, a health effect is frequently associated with protracted exposures of varying intensity sustained in the past. The main complexity of modeling and interpreting such phenomena lies in the additional temporal dimension needed to express the association, as the risk depends on both intensity and timing of past exposures. This type of dependency is defined here as exposure–lag–response association. In this contribution, I illustrate a general statistical framework for such associations, established through the extension of distributed lag non-linear models, originally developed in time series analysis. This modeling class is based on the definition of a cross-basis, obtained by the combination of two functions to flexibly model linear or nonlinear exposure-responses and the lag structure of the relationship, respectively. The methodology is illustrated with an example application to cohort data and validated through a simulation study. This modeling framework generalizes to various study designs and regression models, and can be applied to study the health effects of protracted exposures to environmental factors, drugs or carcinogenic agents, among others. © 2013 The Authors. Statistics in Medicine published by John Wiley & Sons, Ltd.

## 1. Introduction

In biomedical research, it is commonly appreciated that an exposure event produces effects lasting well beyond the exposure period, with an increase in risk occurring from few hours to many years later, depending on the physiological processes linking the exposure and the health outcome. The problem is made even more complicated in the presence of protracted time-varying exposures, when the health effect measured at a given time can be described as the result of multiple exposure events of different intensities sustained in the past. This phenomenon, common to various research fields, has been associated for example with peak 1 or chronic exposures 2 to environmental stressors, drug intake 3,4, or occupational exposures to carcinogenic substances 5.

The main complexity of modeling and interpreting such dependencies lies in the additional temporal dimension needed to express the association, beyond the usual exposure–response relationship, as the risk depends on both intensity and timing of past exposures. Nonetheless, the appropriate representation of the temporal pattern of risks may provide further insights on the association of interest, in particular regarding the underlying pathophysiological mechanisms, and prevent biases in estimates and predictions. Revising previous terminology 6, I define these dependencies as *exposure–lag–response associations*.

In particular, this issue has been debated in cancer epidemiology 7–9. Analytical approaches extend simple indices such as cumulative exposure, in order to accommodate the temporal variation in risk because of protracted exposures. In particular, the pioneering work by Thomas 10,6 helped develop sophisticated statistical methods on the basis of *weighting* past exposures through specific functions whose parameters are estimated by the data. Vacek 11, Langholz and colleagues 12, and Richardson 13 provided interesting applications in case-control studies, with weights represented through simple parametric functions. The methodology was improved by Hauptmann and colleagues in a series of papers 14–16 by using flexible and smooth spline functions. Sylvestre and Abrahamowicz 17 and Abrahamowicz and colleagues 18 extended the spline methods to the analysis of time-to-event data with a cohort design and presented their applications in pharmaco-epidemiology.

The main limitation of the statistical techniques described in these papers is the assumption of a linear exposure–response relationship. Models for nonlinear dependencies introduce further nontrivial complexities, from both statistical and interpretational perspectives, as the problem becomes inherently bidimensional. Abrahamowicz and Mackenzie 19 proposed a model for analyzing the nonlinear time-dependent effects of fixed exposures, while Vacek 11 and Berhane and colleagues 20 extended this scheme to the case of protracted time-varying exposures. However, the modeling techniques illustrated in these other papers still face some limitations, as they are based on complex estimation routines with convergence issues and problems in producing uncertainty measures, such as standard errors and confidence intervals.

Interestingly, equivalent approaches were previously established in time series analysis, on the basis of *distributed lag models* (DLMs), a methodology originally formulated in econometrics 21, then applied in epidemiological research 22. These models involve the definition of a distributed lag function, analogous to the weighting function described before. In particular, Armstrong 23 generalized the method to *distributed lag non-linear models* (DLNMs), a class of models with different options for the functions applied to model nonlinearity and distributed lag effects. The theory of DLMs and DLNMs have been recently re-evaluated 24, offering a well-grounded statistical tool and a comprehensive scheme for interpretation.

In this paper, I aim to establish a general conceptual and statistical framework for modeling exposure–lag–response associations, built upon the paradigm of DLMs and DLNMs. This modeling class, extended beyond time series analysis, provides a unified methodology applicable in different study designs, data structures, and regression models, including most of the previous methods as specific cases. Also, the statistical framework is defined by completely parametric functions and fitted through standard regression methods, with measures of uncertainty and fit statistics easily available. The R package dlnm, originally developed for time series data 25, is extended in parallel, offering a easy-to-use implementation of the modeling approach.

The manuscript is structured as follows. The development and algebraic definition of the modeling framework is described in Section 2. As an illustrative example, in I apply the method for investigating the relationship between occupational exposure to radon and lung cancer mortality by using the data from the Colorado Plateau miners cohort. The modeling framework is then validated in a simulation study n. A final discussion is provided in. Information on data and software implementation is included in. The R code and data are included in the supporting information together with additional details, making the results of the illustrative example and of the simulation study entirely reproducible.

## 2. Modeling framework

The modeling skeleton is derived by extending the class of DLNMs beyond the time series context. This extension provides a neat algebraic representation and a comprehensive statistical definition. The focus is on a function, here defined *s*(*x*,*t*), which describes the dependency in terms of the exposure history to *x* evaluated at time *t*. The function *s*(*x*,*t*) is commonly included in regression models in order to estimate the association, while controlling for potential confounders. Although the regression model varies depending on the study design and the type of data, the definition of *s*(*x*,*t*) provided later and the related modeling framework generally apply.

### 2.1. Models for linear exposure–response relationships

Previous studies on the topic have defined the function *s*(*x*,*t*) by using slightly different algebraic formulae 10,26,11,14,17. Assuming a linear exposure–response relationship, a general notation can be given by


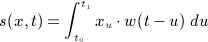
(1a)


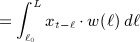
(1b)


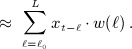
(1c)

In (1a), the increase in risk at time *t* is defined as the integral of the instantaneous exposure intensity *x*_*u*_ over the period Δ*t* = [*t*_0_,*t*_1_], with *t*_0_ and *t*_1_ representing the times of the first and last relevant exposures. Here, *w*(*t* − *u*) is the weighting function previously described in, which assigns weights to past exposures experienced at time *t* − *u* on the basis of their contribution to the risk at time *t*. The model can be reparameterized as in (1b), where the risk is now expressed along the *lag*, with *ℓ* ∈ [*ℓ*_0_,*L*]. Here, *L* − *ℓ*_0_ = *t*_1_ − *t*_0_ is interpreted as the lag period over which an exposure to *x* is assumed to affect the risk at time *t*, usually with *ℓ*_0_ = 0. This parameterization offers the advantage that the function *w* is now directly defined in the new dimension of lag *ℓ*, and it is independent of the time axis chosen for *t*, which may represent different time scales depending on the study design. The function *w*(*ℓ*), termed from here on as the *lag–response function*, models the *lag–response curve* associated with exposure *x*. Finally, for computational purposes, the integral is approximated in (1c) by a sum of terms derived by partitioning the lag interval in equally spaced discrete units and assuming the protracted exposure as a sequence of exposure events *x*_*t* − *ℓ*_ at lags *ℓ* = *ℓ*_0_, …, *L*.

A statistical model for (1) can be defined by expressing the lag–response function *w*(*ℓ*) as a linear combination of terms obtained through basis transformation, with related parameters. By using matrix notation, let the vector **q**_*x*,*t*_ of *exposure history* be defined by


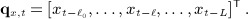
(2)

Such exposure history changes along time, depending on the time *t* at which the vector **q**_*x*,*t*_ is computed. Given (2), the cumulative function *s*(*x*,*t*) in (1) can be written using a compact and general matrix notation as


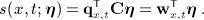
(3)

The (*L* − *ℓ*_0_ + 1) × *v*_*ℓ*_ matrix **C** is obtained from the transformation of the lag vector ***ℓ*** = [*ℓ*_0_, …, *ℓ*, …, *L*]^T^, by choosing a specific basis with dimension *v*_*ℓ*_ for *w*(*ℓ*), which defines the related basis functions. In this parameterization the function *s*(*x*,*t*), representing the integral of *x* · *w*(*ℓ*) over the interval [*ℓ*_0_,*L*], is defined as a *lag–basis* function with parameters ***η***. Interestingly, the equation in (3) is almost identical to that defining DLMs 24, Eq. (4). The different indexing in the original version reflects the specific application in time series, where the data are perfectly ordered in time, and the matrix **Q** has a structure such that *q*_*t*,*ℓ*_ ≡ *q*_*t* + 1,*ℓ* + 1_. However, this is a specific case of the general representation in (2)–(3). The theory and software already developed for DLMs can be therefore extended in parallel.

Alternative lag–basis functions for representing *s*(*x*,*t*) are derived through different lag–response functions *w*(*ℓ*) in (1). In particular the traditional index of unweighted cumulative exposure is a specific case of (3), where 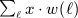 reduces to 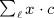 with *w*(*ℓ*) equal to a constant *c*. This is obtained by specifying **C** as an (*L* − *ℓ*_0_ + 1)-dimensional vector of 1's, with *v*_*ℓ*_ = 1. More sophisticated models with splines or other functions, such as those illustrated in publications cited in, only require the application of different bases for deriving **C**, but are nevertheless represented by 3).

### 2.2. Extension to nonlinear exposure–response relationships

The extension to the nonlinear case presents further complexities, as anticipated earlier. The model in (1) can be extended by defining an additional *exposure–response function f*(*x*) to express the potentially nonlinear *exposure–response curve* along the dimension of the predictor. An intuitive generalization of (1) is:


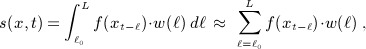
(4)

with *f*(*x*) as the standard exposure–response function. However, the function *f*(*x*) · *w*(*ℓ*) in 4), previously proposed 11,19, is not easily represented as a linear combination of basis variables and generates models that are not linear in their parameters and thus require ad hoc optimization routines. More importantly, this representation is based on the strong assumption of *independency* between *f*(*x*) and *w*(*ℓ*), namely that the exposure–response shape is the same at each lag *ℓ*, and vice versa that the lag structure is the same at each value of *x*. This assumption can be relaxed by expressing *s*(*x*,*t*) as a truly bivariate function, with the more flexible representation:


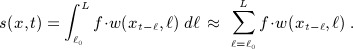
(5)

Here the bidimensional function *f* · *w*(*x*,*ℓ*) is defined as the *exposure–lag–response function*, and models simultaneously the exposure–response curve along *x* and lag–response curve along *ℓ*, namely an *exposure–lag–response surface*.

Differently from 4), the exposure–lag–response function in 5) can be expressed as a linear combination of basis variables and related parameters through a special tensor product. As anticipated earlier, Armstrong 23 proposed the same approach for time series data within the DLNM framework, generalizing this tensor product parameterization through the concept of *cross-basis*. Specifically, two sets of basis functions are independently chosen to represent *f*(*x*) and *w*(*ℓ*), respectively. The cross-basis is the bidimensional space of functions obtained by the combination of the two sets integrated over the lag dimension and represents the core of DLNMs. The algebraic representation has been previously presented 24, and a revised version is proposed here. Briefly, the simpler lag-basis for DLMs in 3) can be extended by choosing an additional basis with dimension *v*_*x*_ for representing *f*(*x*). The application of the related basis functions to the vector of exposure history **q**_*x*,*t*_ obtained by 2) generates a (*L* − *ℓ*_0_ + 1) × *v*_*x*_ matrix **R**_*x*,*t*_. Let **A**_*x*,*t*_ be:


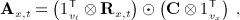
(6)

with **1**_*v*_ as a *v*-dimensional vector of 1's and **C** defined in 3). The cross-basis function *s*(*x*,*t*;***η***) can be defined as


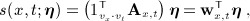
(7)

In this case, the dimension of the cross-basis is determined by the product of the dimensions of the bases for the two spaces, and the association is expressed through *v*_*x*_ · *v*_*ℓ*_ values **W** and related parameters ***η***. The cross-basis function *s*(*x*,*t*) represents the integral of *f* · *w*(*x*,*ℓ*) over the interval [*ℓ*_0_,*L*], cumulating the contributions of events representing the exposure history.

In spite of the relatively complex algebraic form, the definition of cross-basis and the specification of DLNMs only amount to the choice of the bases for the functions *f*(*x*) and *w*(*ℓ*). These can be independently selected between several options such as splines, linear threshold, or piecewise constant (step) functions. The DLNM modeling class comprises the simpler DLMs from Section 2.1. For example, the bidimensional exposure–lag–response function *f* · *w*(*x*,*ℓ*) in 5) reduces to a non-linear function for un-weighted cumulative exposure *f*(*x*) · *c* when *w*(*ℓ*) is a constant function *c*, and to the lag–response function *x* · *w*(*ℓ*) in (1) when *f*(*x*) is simply an linear function of the untransformed *x*. The model proposed by Berhane and colleagues 20 can be written in the form of 6)–7) when both *f*(*x*) and *w*(*ℓ*) are cubic B-splines.

### 2.3. Estimation and prediction

Although the lag-basis and cross-basis functions in (1)–(3) and (5)–(7) involve a nonstandard parameterization in terms of exposure histories, DLMs, and DLNMs do not require specialized estimation procedures. The association is entirely expressed by the *v*_*x*_ × *v*_*ℓ*_ parameters ***η*** of the cross-basis values **W**. The computation of the exposure history in (2) can be extended to all *N* observations with *x* measured at time *t*, producing an *N* × (*L* − *ℓ*_0_ + 1) matrix of exposure histories **Q**. The matrix of transformed variables **W** in (3) and (7) is consequently derived. This matrix can be included in the design matrix of standard regression models to estimate the parameters ***η***. In the completely parametric development proposed here, the number of coefficients *v*_*x*_ × *v*_*ℓ*_ represents the degrees of freedom (*df*) used to model the association.

Inference on the parameters ***η*** and interpretation of the estimated association is aided by the prediction of specific risk measures. For simpler DLMs that assume a linear exposure–response relationship, this step reduces to the computation of a series of estimated risk contributions 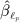 at lag *ℓ*_*p*_, with *ℓ*_0_ *≤ ℓ*_*p*_ *≤ L*, and the associated (co)variance matrix 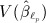. The series of risk contributions 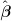 is provided by


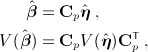
(8)

with **C**_*p*_ obtained from the vector of lag ***ℓ***_*p*_ used for prediction, by applying the same basis functions for *w*(*ℓ*) used for estimation. These estimated risk contributions compose the lag–response curve, and can be interpreted using either a *forward* or *backward perspective*. Namely, 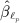 represents the risk contribution at time *t* + *ℓ*_*p*_ in the future from a unit increase in exposure *x* at time *t*, or the contribution from a unit increase in exposure *x* occurring at time *t* − *ℓ*_*p*_ in the past to a given risk measured at time *t*. The estimated risk contributions associated with different exposure increases are easily derived.

The equations in (8) only apply to DLMs with lag-bases as defined in (3). For DLNMs the association is allowed to vary nonlinearly in the space of *x*. Moreover, the specification in (5)–(7) allows the lag-response curve to change depending on the level of the exposure. The prediction of risk contributions 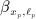 corresponding to a specific exposure intensity *x*_*p*_ at lag *ℓ*_*p*_, involves a more complex procedure. First, let 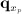 be the (*L* − *ℓ*_0_ + 1)-dimensional vector of exposure history with constant exposure *x*_*p*_. The related matrices 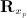 and 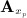 are derived from (6), substituting **q**_*x*,*t*_ and **C** with 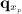 and **C**_*p*_, by applying the same two sets of basis functions for *f* · *w*(*x*,*ℓ*) chosen for estimation. The exposure-specific risk contributions 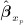 and associated (co)variance matrix 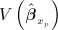 are provided by


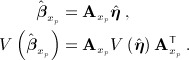
(9)

The estimated risk contributions 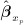 may be interpreted as a lag-response curve similar to 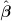 in (8), but this time associated with a specific exposure level *x*_*p*_ instead of a unit increase. These measures may be used to define a grid of predicted risk contributions 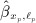 defined within the ranges of the exposure *x* and the lag *ℓ*, thus obtaining a bi-dimensional representation of the association. From this grid, besides 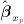 above, it is also possible to derive the vector of lag-specific risk contributions 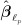, expressing the exposure-response curve for lag *ℓ*_*p*_. As noted in Section 2.2, the truly bivariate definition of (7) allows both the lag-response curve and exposure-response curve, defined by 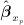 and 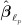 respectively, to change depending on the specific exposure and lag values *x*_*p*_ and *ℓ*_*p*_. The grid is interpreted as a risk surface along *x* and *ℓ* representing the exposure–lag–response.

In addition, predictions in (8)–(9) may be extended to a generic exposure history **q**_*h*_. Substituting it into 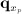 in (9) provides the vector of lag-specific risk contributions 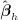 for each exposure that occurred within the lag period. The overall cumulative effect 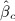 of such exposure history, with associated (co)variance matrix 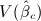 may be computed with:


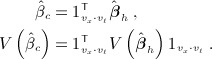
(10)

The Equation (10) can be used to estimate the predicted cumulative risk for a given pattern of exposure **q**_*h*_. This method can also be applied to investigate how the risk progressively evolves along an *exposure profile*, computing the cumulative risk at each time associated with the time-varying exposure history **q**_*h*_.

### 2.4. Identifiability and constraints

The tensor product structure of the cross-basis defined in (5)–(7) poses some identifiability issues. In particular, each of the *v*_*x*_ basis variables in **R** is multiplied by each of the *v*_*ℓ*_ basis variables in **C**. If an intercept is included in *f*(*x*), the related matrix of cross-basis variables **W** is not of full rank, and the parameters of the regression model are not identifiable, even when a common intercept is not included. Therefore the cross-basis in (7) should always be defined without an intercept in the basis functions for *x*. Also, these basis functions can be centered on a specific exposure value *x*_0_, which will represent the reference for the risk summaries computed by (8)–(10).

The bidimensional shape of the exposure–lag–response can be constrained to follow a prespecified pattern. In particular, a priori assumptions on the lag structure can be imposed through functional constraints on the basis for the space of *ℓ*. Left and right constraints on the extremes of the supporting interval *ℓ*_0_–*L* are particularly meaningful for smooth functions. A left constraint can be imposed by excluding the intercept from the basis. This step will force the lag–response curve to predict a null risk at the beginning of the lag period. A right constraint on a B-splines basis can be produced by excluding specific basis variables, as previously described for linear exposure–response relationships 17. The constraint produces a smooth dependency which approaches a null risk at the end of the lag period. Such constraints are particularly useful in the presence of sparse data, in order to limit the flexibility of the model under specific assumptions about the lag–response curve. However, biases can be introduced if these assumptions are not met. Additional information is provided in Section D1 of the supporting information.

The functional constraints discussed in this section can be specified without introducing customized optimization methods for estimating the parameters ***η*** in (3)–(7). More sophisticated methods are required, for example, to constrain the lag–response curve to be non-negative in the whole lag period *L*. These approaches have been previously proposed for linear dependencies 14,17,18 and introduce further complexities in the bidimensional context of DLNMs. This development is not pursued here.

### 2.5. Model selection and inferential procedures

The framework described in Sections 2.1–2.2 includes a fairly large number of models, defined by different functions for each of the two dimensions and by different choices regarding each function, such as number and location of knots in splines. This raises the issue of selecting the optimal model for describing the exposure–lag–response association. Previous studies on temporal dependencies have proposed selection procedures on the basis of profile likelihood 15, AIC 14,16,20 or BIC 17. Simulation studies seems to indicate a better performance of AIC when compared with BIC in this context 18, a result consistent with unpublished simulations performed on time series data for DLNMs.

Inference on the models illustrated in the previous sections primarily focuses on the specification of confidence intervals for the risk measures in Section 2.3 and on the definition of tests for a set of null hypotheses. Confidence intervals for lag–response curves, exposure–response curves and cumulative risks obtained through 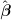, 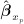, 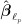 and 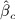 can be easily derived from the diagonal of the related (co)variance matrices in (8)–(10), assuming a multivariate normal distribution of the estimators. Regarding hypothesis testing, two null hypotheses are particularly relevant in this framework. The first one postulates a linear exposure–response relationship, namely *H*_0_ : *f*(*x*) = *x*. The second one assumes a constant risk, namely *H*_0_ : *w*(*ℓ*) = *c*. Tests on constrained models can be also defined. The assumption of independency is not easily tested, as the form in (4) cannot be expressed as a model linear in its parameters.

However, defining general inferential procedures in this setting is not straightforward. First, the null hypotheses *H*_0_ : *f*(*x*) = *x* and *H*_0_ : *w*(*ℓ*) = *c* are not independent, and an incorrect assumption about the association in one dimension may bias the test estimator for the hypothesis related to the other space, as previously reported 19. In addition, estimates are usually conditional on a posteriori selection of a best-fitting model, based on the selection methods discussed before. Under these conditions, the estimators for the (co)variance matrices in (8)–(10) are likely to underestimate the true sampling (co)variance, and the distribution of the test statistics may be different from that assumed unconditional on the selection procedure. This may generate undercoverage of confidence intervals and inflated type I error for tests 17,27.

Given these complexities, a general framework for hypothesis testing embedded in the model selection procedure is not provided here. An assessment through simulations of the performance of estimators generated by AIC and BIC-selected models will be presented in. Specifically, simulations will provide an empirical evaluation of the ability of the information criteria to identify the correct model between those defining the null or alternative hypotheses about linearity and constant effects, and measures of performance such as bias, coverage, and root mean square error.

## 3. An application

The conceptual and statistical framework of DLNMs described in, extended beyond time series data, is general and applicable in different study designs. As an illustrative example, I propose here an application in survival analysis of time-to-event data. This represents one of the most complex settings, as the temporal pattern of risk is produced by exposure histories that vary during the follow-up of each subject. Specifically, the methodology is used to investigate the association between occupational exposure to radon and mortality for lung cancer. The analysis is based on data from the Colorado Plateau uranium miners cohort, already used in previous methodological contributions 12,15,20. Section A of the supporting information provides a list of the main steps to replicate the analysis in other real-life examples.

### 3.1. Data

The cohort data used in this example were collected by the National Institute for Occupational Safety and Health. Detailed information on the cohort is given elsewhere 12. Briefly, subjects were eligible to enter the cohort if they worked in mines within the Colorado Plateau area between 1950 and 1960, and provided demographic, personal and occupational information during their working period. Vital status and cause of death were ascertained by linkage with different sources. The data used in this example refer to the follow-up of the cohort on December 31, 1982, including 3347 subjects and 258 lung cancer deaths. Exposure data available in the data set include cumulative measures of radon and smoking in 5-year age intervals. The radon exposure history for each subject, expressed in working-level months (WLM), was reconstructed by linking employment information with measured or predicted levels in each mine in each year. The smoking history, expressed in the number of cigarettes packs × 100, was reported by each subject during his working period and assumed constant after the last reporting age. A summary of the data is provided in Table I.

**Table I:** Descriptive statistics of the Colorado Plateau uranium miners cohort. The data included here refer to the follow-up on December 31, 1982. Exposure to radon is measured in working level months (WLM), while smoking is reported as packs of cigarettes/100

	Full cohort					Lung cancer cases				
		
		*N*		%			*N*		%	
Subjects		3347		100.0			258		7.7	
Deaths (%)		1258		37.6			258		100.0	
Ever smokers (%)		2656		79.4			238		92.2	

	Median	Min	25th	75th	Max	Median	Min	25th	75th	Max
Age at entry	34.0	15.8	25.8	44.0	80.0	41.6	18.6	34.3	48.0	63.9
Follow-up time (years)	23.9	0.1	19.6	25.5	32.5	18.3	0.3	12.9	22.0	30.8
Exposure to radon										
Exposure period (years)	6.7	0.1	2.7	11.8	53.0	12.8	0.1	7.8	17.6	39.5
Total cumulative exposure (WLM/year)	429.0	0.0	153.5	1016.8	10,000.0	1231.9	8.0	553.7	2528.6	10,000.0
Yearly exposure (WLM/year)										
All	60.2	0.1	26.7	122.2	3245.3	81.6	1.0	42.3	165.4	1295.7
Lag 0–9	52.4	0.1	23.8	102.5	2994.0	61.4	3.9	31.3	144.7	1110.8
Lag 10–19	53.8	0.1	24.3	112.5	3245.3	78.3	1.0	42.9	164.0	1295.7
Lag 20–29	74.0	0.1	33.0	141.7	3245.3	104.7	4.1	52.2	180.0	1295.7
Lag 30–40	95.7	0.2	48.0	151.6	2994.0	104.7	5.5	60.0	175.3	860.2
Smoking										
Exposure period (years)	38.0	5.0	31.0	46.0	75.0	40.0	14.0	33.0	48.0	72.0
Total cumulative exposure (packs × 100)	131.6	0.4	94.5	174.5	676.3	147.4	21.8	109.5	188.1	567.2
Yearly exposure (packs × 100)	3.6	0.0	2.5	3.6	24.4	3.6	0.0	3.5	4.2	13.4

### 3.2. Modeling strategy

For this illustrative example, the analysis is performed through a Cox proportional-hazard model with time-varying covariates by using age as the time axis. Effect measures are reported as a hazard ratio (HR). The model is represented by the following:


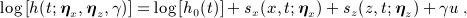
(11)

where the log-hazard log [*h*(*t*)] is expressed as a sum of baseline log-hazard log [*h*_0_(*t*)] and contributions of additional covariates. These comprise cross-basis functions *s*_*x*_(*x*,*t*) and *s*_*z*_(*z*,*t*) for radon and smoking respectively, as defined in (1)–(7), and a linear term for calendar time *u*, in order to control for secular trends in lung cancer risk not accounted for by the delayed effects of the two exposures. Radon is the exposure of interest and is modeled with different combinations of bases for *f*(*x*) and *w*(*ℓ*) in the cross-basis *s*_*x*_(*x*,*t*). Given the limited information on smoking histories, in this analysis, the cross-basis *s*_*z*_(*z*,*t*) is a priori defined with a natural cubic B-spline with one knot at the median of 2.5 yearly packs × 100 for the exposure–response and a step function with a single cut-off at lag 20 for the lag structure, with lag period 2–40 years. However, different cross-basis functions can be applied. The model spends 5 *df* controlling for confounders, and a different amount for modeling the effect of radon, depending on the chosen cross-basis *s*_*x*_(*x*,*t*).

Modeling exposure–lag–response associations in time-to-event data assumes the definition of an extended version of continuous time-varying predictors, namely the varying exposure history for each subject at the ages he contributes to different risk sets 28. The lag scale is chosen as years, with lag 0 identifying the exposure during the last year. The lag period is fixed at 2–40, assuming no effect of exposure after 40 years and in the last 2 years, consistently with previous analyses. Multiple exposure histories are computed for each subject at the ages he contributed to each risk set, given his exposure profile reconstructed from the 5-year periods. This step produced matrices of exposure histories **Q**_*x*_ and **Q**_*z*_ for radon and smoking respectively, as defined in (2). These matrices are used to specify the lag-bases or cross-bases matrices **W**_*x*_ and **W**_*z*_ from (3)–(7), included in the design matrix of the Cox model. Additional information is provided in the Section B of the supporting information.

The functions *f*(*x*) and *w*(*ℓ*) composing *s*_*x*_(*x*,*t*) for the model candidates are selected a priori among linear, constant, piecewise constant functions, and quadratic B-splines, with 36 models in total. Specifically, the three cut-offs of a piecewise constant function and combinations of 0, 1, or 2 knots for B-splines are placed at quartiles for the dimension of *x*, corresponding to 26.7, 60.2, and 122.2 WLM/year, and at 13.3, 20, or 26.6 lags for the dimension of *ℓ*. Also, in alternative parameterizations of *w*(*ℓ*), the intercept is excluded in the B-spline bases, left-constraining the smooth lag–response curve to start from a null risk at lag 2. This a priori assumption reasonably follows the hypothesis that the risk associated with past exposures smoothly increases from zero starting from lag 2. Model selection is based on AIC and BIC adapted to survival analysis, given by the following:


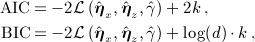
(12)

where 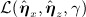 is the log-likelihood of the fitted model, *k* is the number of total *df*, and *d* is the number of uncensored events. The best-fitting model (11) is chosen by minimizing AIC or BIC in (12). Both criteria apply a multiplicative constant to the number of parameters for penalizing more complex models. In particular, the penalty of BIC (equal to log(*d*)) is usually higher and tends to select simpler models.

### 3.3. Results for distributed lag models

Results for simple DLMs, assuming a linear radon–mortality relationship on the log scale, are illustrated first. Table II presents models with different functions *w*(*ℓ*), as defined in (3). Specifically, model 1 is specified by a constant (intercept only) function, producing a lag-basis identical to the traditional index of unweighted cumulative exposure; model 2 is an example of a DLM with a piecewise constant function; the best-fitting B-spline models with and without intercept, specified by a single knot at 13.3 lags are reported as models 3 and 4, respectively. The fit of the various options is expressed by AIC and BIC, with the best performance achieved by model 1 for both criteria. This model assigns the same importance to the exposures experienced *ℓ* lags earlier in defining the risk for a given time. The specification of more flexible functions with more *df* does not seem to improve the fit.

**Table II:** Functions *f*(*x*) and *w*(*ℓ*), total degrees of freedom (*df*) associated with the cross-basis, and values for the AIC and BIC for alternative models for the exposure–lag–response association between radon and mortality. Data from the Colorado Plateau uranium miners cohort

DLMs					
		
	*f*(*x*)	*w*(*ℓ*)	*df*	AIC	BIC
Model 1	Linear	Constant	1	2236.0	2257.3
Model 2	Linear	Piecewise constant†	4	2238.6	2270.6
Model 3	Linear	Quadratic B-Spline‖	4	2238.8	2270.8
Model 4	Linear	Quadratic B-Spline§	3	2238.9	2267.3

DLNMs					
		
	*f*(*x*)	*w*(*ℓ*)	*df*	AIC	BIC
Model 5	Quadratic B-Spline*	Constant	3	2181.4	2209.8
Model 6	Piecewise constant‡	Piecewise constant†	12	2171.6	2232.0
Model 7	Quadratic B-Spline*	Quadratic B-Spline‖	12	2155.3	2215.7
Model 8	Quadratic B-Spline*	Quadratic B-Spline§	9	2153.2	2202.9

‡ Cut-offs at 26.7, 60.2, and 122.2 WLM/years.

† Cut-offs at 10, 20, and 30 lag.

* Knot at 60.2 WLM/years.

∥ Knot at 13.3 lag.

§ Knot at 13.3 lag, no intercept.

DLM, distributed lag models; DLNMs, distributed lag non-linear models.

**Figure 1:**
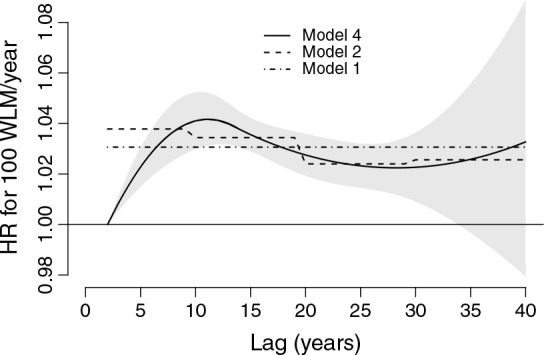
Hazard ratio (HR) of lung cancer mortality associated with radon exposure to 100 WLM/year in the lag period of 0–40 years. The figure shows the lag–response curves estimated from models 4 (with 95%CI), 2, and 1, as specified in Table II. Data from the Colorado Plateau uranium miners cohort.

Figure 1 shows the lag–response curves estimated from models 1, 2, and 4. The curves are composed of a series of estimated contributions 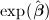 to the risk of mortality for lung cancer at each lag *ℓ*, associated with an increase of 100 WLM/year in radon exposure, with 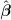 defined in (8). The results can be interpreted following the scheme described in Section 2.3. By using a forward perspective, 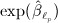 represents the HR contribution from a unit increase in exposure experienced at *t* to the subsequent risk at *t* + *ℓ*, with *ℓ* = 2, …, 40 years. Alternatively, adopting a backward perspective, the same summary is interpreted as the HR contribution from a unit increase in exposure experienced at *t* − *ℓ* to the overall risk at *t*. Model 4 predicts a maximum increase in risk at lag 11, with a HR of 1.042 (95%CI: 1.031–1.052), compared with the constant HR of 1.031 (95%CI: 1.025–1.036) estimated from model 1.

The better performance of model 1 seems to indicate that the hypothesis of constant risk *H*_0_ : *w*(*ℓ*) = *c* is supported by the data. Also, the lag–response curve from model 4 in Figure 1 does not suggest a decrease in risk at longer lags, although the confidence intervals are relatively wide in this part of the lag period.

### 3.4. Results for distributed lag non-linear models

The results illustrated in Section 3.3 are dependent on the strong assumption of a log-linear relationship between radon exposure and lung cancer mortality. The analysis can be repeated with more flexible DLNMs, which can describe simultaneously nonlinear exposure–response relationships and lag structures through the specification of a cross-basis in (5)–(7). The definition of DLNMs involves a higher number of potential models, obtained by different combinations of bases for the functions *f*(*x*) and *w*(*ℓ*). The second part of Table II only reports models with the same choices for *w*(*ℓ*) used in DLM, and with *f*(*x*) specified as another piecewise constant function and the B-spline providing the best fit, with one knot at 60.2 WLM/year. Overall, the best-fitting option for both AIC and BIC is model 8, with a B-spline for both *f*(*x*) and *w*(*ℓ*). This model uses 9 *df* in total for expressing the bidimensional association.

The hypothesis *H*_0_ : *f*(*x*) = *x* of a linear radon-mortality dependency is not supported by the data, as all the DLNMs show a substantial decrease in both AIC and BIC, when compared with simpler DLMs. In particular, the comparison of the best-fitting model 8, representing *f* · *w*(*x*,*ℓ*), with model 4, representing *x* · *w*(*x*,*ℓ*), indicates that the 6 additional *df* substantially improve the fit. Similarly, the hypothesis *H*_0_ : *w*(*ℓ*) = *c* of a constant risk along lags, previously suggested when evaluating DLMs, is not supported either. The comparison of model 8 with model 5, representing *f*(*x*) · *c*, indicates a better fit of the former. Interestingly, this result is the opposite of what was suggested in Section 3.3, revealing how imposing a wrong assumption about the relationship in one dimension induces spurious results in the other space, compromising the analysis of the association.

**Figure 2:**
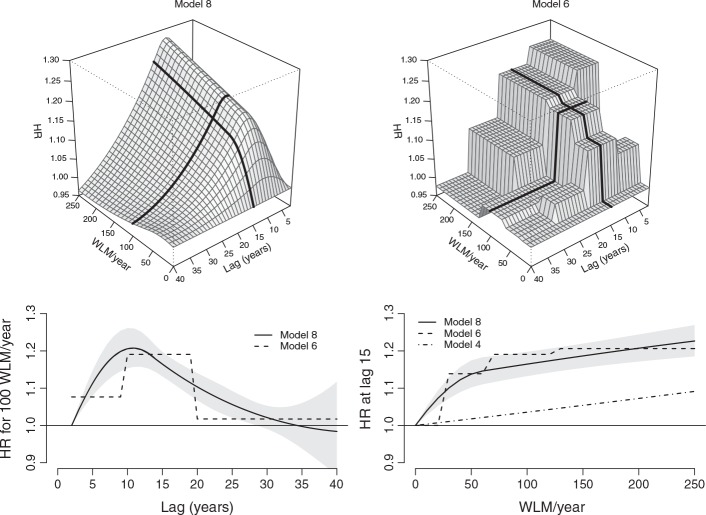
Hazard ratio (HR) of lung cancer mortality associated with radon exposure in the range 0–250 WLM/year and lag period 0–40 years. The figure shows 3-D graphs of the exposure–lag–response association on a grid of exposure × lag values (from model 8, top left, and model 6, top right), lag–response curves for radon exposure of 100 WLM/year (from models 8, with 95%CI, and model 6, bottom left), and exposure–response curves at lag 15 (from models 8, with 95%CI, and models 6 and 4, bottom right). The models are described in Table II. Data from the Colorado Plateau uranium miners cohort.

The interpretation of results from DLNMs relies on a bidimensional representation of the exposure–lag–response association. This is achieved by computing the risk contributions 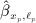 over a grid defined in the range of the exposure *x* and the lag *ℓ*, applying (9). This bidimensional dependency is depicted in the two top panels of Figure 2, showing the predicted HR surfaces from models 8 and 6, in the range 0–250 WLM and 0–40 lags. The graphs show an initial increase in risk along lags, peaking at approximately 10 years after the exposure, and then decreasing and apparently disappearing after about 30 years, independent of the exposure levels. The inspection of the panels along the dimension of *x* reveals the nonlinear radon-mortality dependency, with the risk increasing steadily up to 50 WLM/year, and then flattening out. The shape of the HR surfaces unveils the different assumptions underlying the choices of bases for functions *f*(*x*) and *w*(*ℓ*), namely B-splines and piecewise constant functions.

Although the 3-D representation offered by the top panels in Figure 2 provides an overview of the bidimensional association, it is still limited for inferential purposes, as the uncertainty in the estimate is not reported. In order to extend the interpretation, the analysis can focus on the risk along *ℓ* predicted for specific exposure intensities, or alternatively the risk along *x* for specific lags, namely the vectors 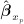 and 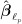 from Section 2.3. These dependencies are represented by slices cut on the bidimensional risk surface along the appropriate dimension. The bottom panels of Figure 2 report the lag–response curve corresponding to an exposure level of 100 WLM/year and the exposure–response curve for lag 15 from both Models 8 and 6, together with 95% confidence intervals for the former. These curves correspond to the two bold lines in the 3-D plots.

**Figure 3:**
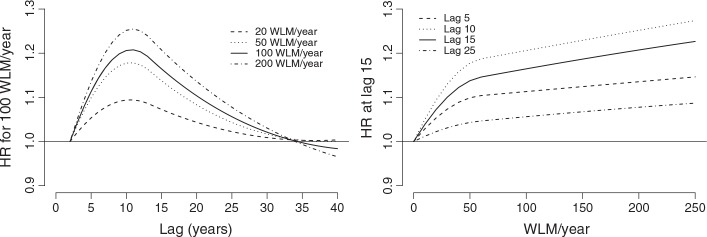
Hazard ratio (HR) of lung cancer mortality associated with radon exposure in the range 0–250 WLM/year and lag period 0–40 years. The figure shows lag–response curves for radon exposure of 20, 50, 100, and 200 WLM/year (left), and exposure–response curves at lag 5, 10, 15, and 20 (right), estimated from model 8, as specified in Table II. Data from the Colorado Plateau uranium miners cohort.

The bottom-left panel is interpreted similarly to the DLM in Figure 1 as the specific risk contributions 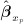 composing the lag–response curve, but this time associated with a specific exposure *x*_*p*_ = 100 WLM/year. The curve estimated from model 8 peaks at lag 11, with an HR of 1.21 (95%CI: 1.16–1.26), and both models 8 and 6 suggest that the risk disappears after 30–35 years. The B-spline for *w*(*ℓ*) in model 8 is left-constrained by the lack of an intercept, forcing the smoothed lag–response curve to start from a null risk at lag 2. The best fit of Model 8 if compared with model 7, which includes the intercept, seems to support this hypothesis. The bottom-right panel shows instead the risk contributions 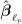 at *ℓ*_*p*_ = 15 for different exposure intensities and is interpreted as the exposure–response at *t* from exposures experienced at *t* − 15 (backward perspective), or the exposure–response contributions at *t* + 15 from exposures experienced at year *t* (forward perspective). Although models 8 and 6 adopt different bases for functions *f*(*x*) and *w*(*ℓ*), the estimates of the predicted risk along *x* and *ℓ* are consistent, showing a radon-mortality relationship that is markedly nonlinear and nonconstant in time. These measures of risk are extended in Figure 3, reporting estimates from model 8 for different exposure and lag values. A right-constrained version of Model 8 is discussed in Section D.1 and illustrated in Figure S2 of the supporting information.

Interestingly, even though model 8 is produced from the flexible definition in (5)–(7), Figures 2 and 3 suggest that the assumption of independency holds here, with shapes of the exposure-response and lag-response curves at different values of *ℓ*_*p*_ and *x*_*p*_ respectively being proportional, and the maximum HR constantly experienced at lag 11. This result reinforces the fact that the cross-basis representation, based on a truly bivariate exposure–lag–response function *f* · *w*(*x*,*ℓ*), may appropriately describe the specific independency case defined by the simpler representation *f*(*x*) · *w*(*ℓ*) in (4).

### 3.5. Prediction for specific exposure histories

The flexible modeling approach described here can be applied to predict the overall cumulative risk 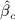 from (10) for a specific exposure history **q**_*h*_, as outlined in Section 2.3. Table III illustrates the predicted HR from four different models in five alternative exposure scenarios. This approach, previously proposed 17, provides clear and interpretable risk summaries from complex models in the presence of varying exposure patterns. The first two scenarios refer to a constant radon exposure of 20 and 100 WLM/year, respectively, in the past 10 years. As expected, simple DLMs (models 1 and 4) predict a similar risk, but substantially lower than the two DLNMs with a B-spline for *f*(*x*). In particular, model 5 extends model 1 by allowing a nonlinear dependency for the unweighted cumulative exposure, estimating a slightly lower risk when compared with the more flexible model 8 already described. The third scenario extends the exposure to 20 WLM/year in the previous 20 years, while the fourth one assumes that a 10-year exposure to the same intensity ceased 10 years before. The comparative assessment of the four models is similar to the first two examples. The last scenario considers the risk of more remote exposures, occurring 30–39 years ago. Interestingly, models 1 and 5 provide identical estimates to the fourth scenario, as the risk of past exposures is assumed constant along the whole lag period. Model 8 instead predicts no excess in lung cancer in the last scenario, given at least 30 years passed from the last exposure to radon, a lag period for which the lag–response curve in Figure 2 (bottom-left panel) displays a null risk.

**Table III:** Overall cumulative hazard ratio (with 95%CI) of lung cancer mortality associated with alternative scenarios of exposure histories to radon, as predicted from models 1, 4, 5, and 8, described in Table II

	Model 1	Model 4	Model 5	Model 8
				
Exposure scenario	*x* · *c*	*x* · *w*(*x*,*ℓ*)	*f*(*x*) · *c*	*f* · *w*(*x*,*ℓ*)
20 WLM/year in the last 10 years	1.05 (1.04–1.06)	1.04 (1.03–1.05)	1.33 (1.22–1.46)	1.52 (1.31–1.76)
100 WLM/year in the last 10 years	1.27 (1.22–1.33)	1.20 (1.13–1.27)	1.96 (1.73–2.22)	2.37 (1.87–2.99)
20 WLM/year in the last 20 years	1.11 (1.09–1.14)	1.11 (1.09–1.14)	1.92 (1.56–2.35)	3.12 (2.29–4.24)
20 WLM/year 10–19 years ago	1.06 (1.05–1.07)	1.07 (1.06–1.09)	1.43 (1.28–1.61)	2.05 (1.70–2.48)
20 WLM/year 30–39 years ago	1.06 (1.05–1.07)	1.05 (1.00–1.11)	1.43 (1.28–1.61)	1.04 (0.64–1.70)

WLM, working-level months.

**Figure 4:**
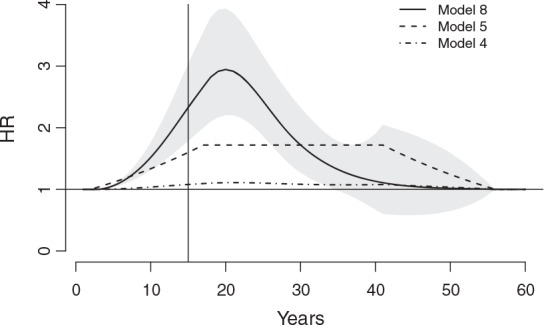
Trend of hazard ratio (HR) of lung cancer mortality in the period 1–60 years associated with radon exposure of 20 WLM/year experienced in years 1–15, predicted from models 8 (with 95%CI), 5, and 4, as specified in Table II.

The summaries illustrated in this section can be extended to predict how the risk evolves dynamically in time in association with time-varying exposures. Adopting a forward perspective, the risk changes along an exposure profile, with specific exposures events referring to different lags and producing a different exposure history. As an example, Figure 4 displays the overall cumulative mortality risk within years 0–60 for an exposure to 20 WLM/year experienced in the first 15 years. Here, model 8 predicts an HR peak of 2.94 (95%CI: 2.20–3.93) at around 20 years, 5 years after the end of the exposure. The plot also suggests that model 4, assuming a log-linear exposure–response relationship, seriously underestimates the risk of lung cancer for four decades, predicting an HR at year 20 of 1.11 (95%CI: 1.09–1.13). Also, the assumption of a constant risk along lags of a nonlinear relationship, adopted in model 5, produces an underestimation of the predicted HR in the first part of the period, followed by a clear overestimation in the last years.

### 3.6. On linearity and the ’nonspecial’ case of log transformation

The bottom-right panel of Figure 2 reports the exposure–response for the simple DLM in model 4, which predicts a substantially lower risk than the two DLNMs. This difference is also evident when comparing the HR range in Figures 1 and 2 (bottom-left panel). This discrepancy is related to the wrong assumption of a linear radon-mortality dependency, with the fit of model 4 highly dependent on a few of very high exposure occurrences. A sensitivity analysis performed on the subset of subjects with a maximum yearly exposure to radon of less than 300 WLM/year (81.6% of the total) is illustrated in Section D.2 of the supporting information.

The highly skewed distribution of exposure events to radon and the shape of the estimated exposure–response curve suggest a log transformation of the exposure. In fact, this model can be still described as a DLNM in (5)–(7), characterized by a basis with dimension *v*_*x*_ = 1 corresponding to *f*(*x*) = log(*x* + 1). A new model is defined by replacing the spline in model 8 with the log function. The comparison is presented in details in Section D.3 of the supporting information. Although this more parsimonious model slightly improves the fit, with an AIC of 2148.6, it is worth noting that results are very similar, as illustrated in Figure S4 (supporting information), suggesting that the spline function is flexible enough to recover the association. More generally, different functions than those presented here can be used to define the exposure–response or lag structure.

## 4. Simulation study

The performance of the extended DLNM framework is validated through simulations, under different scenarios of exposure–lag–response associations. Specifically, the framework is evaluated by estimating the relative bias, coverage and relative root mean square error (RMSE) of the estimators derived from AIC and BIC selection, and the empirical rejection rates for the hypotheses *H*_0_ : *f*(*x*) = *x* and *H*_0_ : *w*(*ℓ*) = *c* of linearity and constant effects, respectively.

### 4.1. Simulation design and data generation

The simulation setting involves the generation of exposure profiles for a set of *n*_*s*_ subjects, the definition of scenarios with known bidimensional exposure–lag–response associations, and the random generation of time-to-event occurrences from such scenarios. These steps are briefly summarized here, with more detailed information provided in Section E of the supporting information.

**Figure 5:**
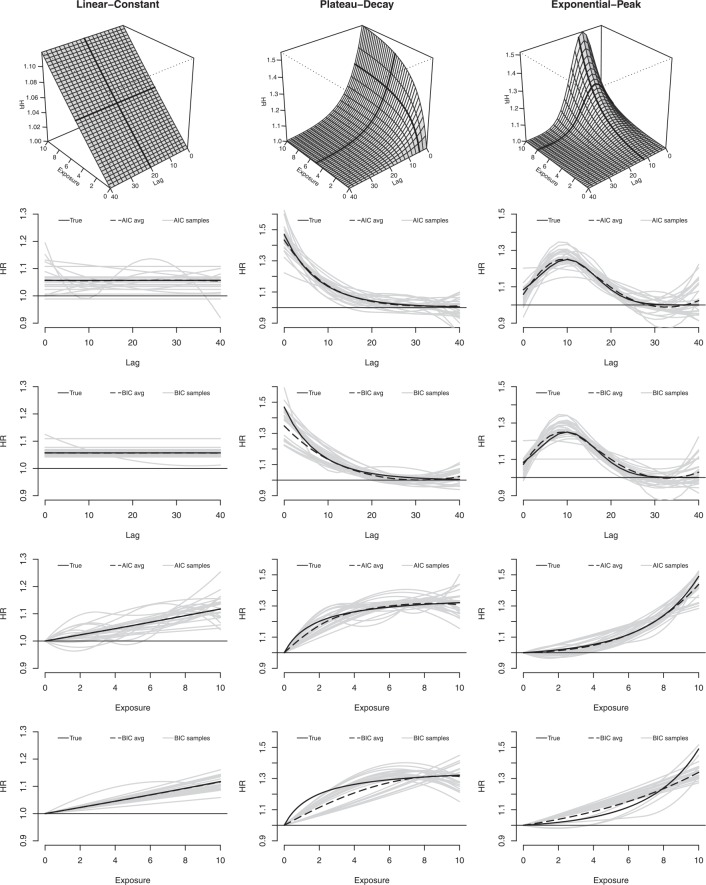
Results of the simulation study for three scenarios of exposure–lag–response associations (linear-constant, plateau-decay, and exponential-peak, in each column). The graphs illustrate the true simulated 3-D exposure–lag–response association (first row), and the lag–response (second–third rows) and exposure–response curves (fourth–fifth rows) from AIC and BIC-selected models, corresponding to the bold lines in the 3-D plots. These last panels compare the true simulated associations with the average of the estimated associations, together with a sample of estimated curves corresponding to the first 25 simulations (in grey). Results from *m* = 500 simulated data sets with *n*_*s*_ = 400 subjects.

The time-varying exposure profiles for *n*_*s*_ subjects are represented as series of occurrences *x*_*t*_ at time *t* = 1, …, 100, generated by random exposure events with an intensity in the range 0–10. The exposure–lag–response associations are defined by the function *f*_*s*_(*x*) · *w*_*s*_(*ℓ*) in (4), which is simpler to simulate if compared with the truly bivariate alternative in (5), for each value of exposure *x* and lag *ℓ*. Different scenarios explore alternative choices for the exposure–response function *f*_*s*_(*x*) and the lag–response function *w*_*s*_(*ℓ*). These are obtained by simple mathematical functions involving logarithms or exponentials. Specifically, *f*_*s*_(*x*) is specified as *linear*, *plateau*, and *exponential*, while *w*_*s*_(*ℓ*) as *constant*, *decay*, and *peak* (see Figure S7 in the supporting information). Three scenarios out of the nine possible combinations are shown in the top panels of Figure 5, the others in Figure S8 (supporting information).

Time-to-event data are simulated conditional on the cumulative contribution of the simulated exposure, using a permutational algorithm previously proposed for time-varying exposures 29. The cumulative effect is calculated in the form of a function 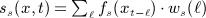 defined in (4), given the exposure history of each subject at time *t*, over a lag period 0–*L*, with *L* = 40. Censoring events are included and represent approximately 25% of the total. For each of the nine scenarios, *m* = 500 data sets are simulated with a number of subjects *n*_*s*_ equal to 200, 400, or 800.

### 4.2. Evaluation of performance

For each data set *i* = 1, …, 500, the exposure–lag–response association is estimated by Cox regression models with a cross-basis 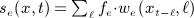 as defined in (5)–(7). The Efron method is used for tie handling. Similarly to the example in, the exposure–response function *f*_*e*_(*x*) is specified as a simple linear term, or quadratic B-splines with 0,1, or 2 knots placed at 3.3, 5, or 6.7. The lag–response function *w*_*e*_(*ℓ*) is specified as a simple constant term with 1 *df*, or quadratic B-splines with intercept and 0, 1, or 2 knots placed at 13.3, 20, or 26.7 lags. The total number of *df* for the cross-basis function *s*_*e*_(*x*,*t*) ranges from 1 × 1 = 1 to 4 × 5 = 20 in the 36 models. For each simulated data set, the best-fitting models are selected as those minimizing AIC and BIC in (12), respectively.

Performance is formally evaluated using a synthetic risk summary *β*_*c*_ from (10), corresponding to an overall cumulative effect, and then visually assessed on the whole exposure–lag–response association. The formal evaluation consists in the computation of different *β*_*c*,*i*_ at each *i*^*th*^ iteration, given an exposure history **q**_*h*,*i*_ evaluated at a random time *t* between 41 and 100 for a random individual among the *n*_*s*_ subjects. Indices of relative bias, coverage and relative RMSE are derived from the following:


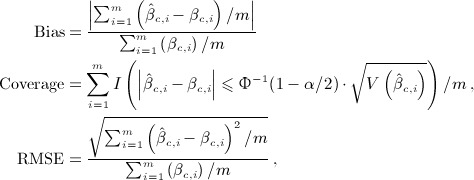
(13)

where *I* is an indicator function, and Φ^− 1^(1 − *α*) is the quantile function of the cumulative normal distribution related to probability 1 − *α*, with *α* = 0.05. The effect summary *β*_*c*,*i*_ corresponds to the true effect from 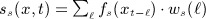, while 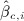 is estimated from the best fitting model selected by AIC and BIC, by using 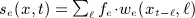, given the specific exposure history **q**_*h*,*i*_ of the random subject at the random time. This approach assures that the performance indicators in (13) are evaluated on the whole range of simulated exposure histories, and do not depend on a specific choice. A visual inspection of performance is also provided by computing from the best-fitting models the grid of risk contributions 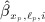 defined in (9) composing the exposure–lag–response surface. Bias is then assessed across the surface by comparing the average fit of the the *m* = 500 models with the true exposure–lag–response relationship. A bidimensional display of coverage is also provided for each scenario.

The performance of the AIC and BIC are also evaluated through their empirical rejection rate for the hypotheses of linearity or constant effect, namely the proportion of times the selection procedure favors a model with a non-linear term for *f*_*e*_(*x*) and a non-constant term for *w*_*e*_(*ℓ*). When *H*_0_ is true, namely *f*_*s*_(*x*) = *x* or *w*_*s*_(*ℓ*) = *c*, the rejection rate is an estimate of the probability of error of the selection criteria, which wrongly select unnecessarily complex models. When *H*_0_ is false, namely *f*_*s*_(*x*) ≠ *x* or *w*_*s*_(*ℓ*) ≠ *c*, the rejection rate is an estimate of the power of the selection criteria for identifying non-linearity and constant lag structures. In a formal hypothesis testing setting, these measures would be interpreted as the type I error and the power of the test.

### 4.3. Results of the simulation study

The results of simulations under the nine scenarios with *n*_*s*_ = 400, producing approximately 300 uncensored events, are summarized in tables in graphs. Table IV reports the formal evaluation of performance on the synthetic risk summary *β*_*c*_, in terms of relative bias, coverage, and relative RMSE. A visual assessment for three scenarios is provided in each column of the multi-panel Figure 5. The true simulated exposure–lag response associations are displayed in the top panels, while the other panels offer a comparison of the true lag–response and exposure–response curves at specific values with the average of the estimates from AIC and BIC-selected models, together with a sample of 25 individual curves.

**Table IV:** Synthetic indices of relative bias, coverage and relative root mean square error (RSME) for the nine scenarios of exposure–lag–response associations. Results from *m* = 500 simulated data sets with *n*_*s*_ = 400 subjects

	Bias		Coverage		RMSE	
			
*f*(*x*) − *w*(*ℓ*)	AIC	BIC	AIC	BIC	AIC	BIC
Linear-constant	0.01	0.01	0.91	0.94	0.07	0.04
Linear-decay	0.00	0.00	0.93	0.94	0.07	0.05
Linear-peak	0.01	0.01	0.92	0.90	0.08	0.07
Plateau-constant	0.06	0.13	0.84	0.72	0.08	0.09
Plateau-decay	0.04	0.14	0.90	0.74	0.09	0.13
Plateau-peak	0.07	0.21	0.87	0.62	0.11	0.18
Exponential-constant	0.01	0.03	0.90	0.80	0.09	0.09
Exponential-decay	0.05	0.04	0.93	0.87	0.12	0.13
Exponential-peak	0.00	0.17	0.91	0.75	0.12	0.17

Generally, AIC-selected models offer a better performance, with a lower relative bias and a coverage of confidence intervals closer to the 95% nominal value. The values of relative RMSE suggest that the higher variability of AIC-based estimators is often balanced by the higher bias affecting BIC. At least part of the bias can be attributed to lack of fit, due to the insufficient flexibility of quadratic spline functions when used to fit logarithmic or exponential shapes. This phenomenon appears quite relevant for the plateau-type exposure response, characterized by the highest relative bias, in the order of 4–7% for AIC but up to 21% for BIC (see Table IV and Figure 5, second column). This pattern is confirmed by the results in Table V, showing the average *df* in each dimension and the empirical rejection rates for the hypotheses of linearity and constant risk. The AIC selection is affected by moderate overfitting, sometimes suggesting flexible models in scenarios of linear and/or constant risk. In contrast, BIC shows severe underfitting, often selecting simple models for complex exposure–lag–response associations, in particular regarding linearity.

**Table V:** Average *df* in each dimension for the best fitting models selected through AIC and BIC (left part), and empirical rejection rate for the AIC and BIC-based selection for the hypotheses of linearity and constant risk (right part) for the nine scenarios of exposure–lag–response associations. Results from *m* = 500 simulated data sets with *n*_*s*_ = 400 subjects

	Average *df*				Empirical rejection rate			
		
	*f*(*x*)		*w*(*ℓ*)		*H*_0_ : *f*(*x*) = *x*		*H*_0_ : *w*(*ℓ*) = *c*	
				
*f*(*x*) − *w*(*ℓ*)	AIC	BIC	AIC	BIC	AIC	BIC	AIC	BIC
Linear-constant	1.50	1.03	1.57	1.02	0.29*	0.03*	0.23*	0.01*
Linear-decay	1.26	1.00	3.60	3.17	0.18*	0.00*	1.00	1.00
Linear-peak	1.22	1.00	4.02	3.72	0.15*	0.00*	1.00	0.98
Plateau-constant	2.26	1.54	1.47	1.00	0.82	0.47	0.19*	0.00*
Plateau-decay	2.53	1.55	3.49	3.10	0.97	0.54	1.00	1.00
Plateau-peak	2.18	1.21	4.01	3.56	0.85	0.19	1.00	0.93
Exponential-onstant	2.20	1.56	1.43	1.00	0.83	0.52	0.16*	0.00*
Exponential-decay	2.36	1.81	3.58	3.12	0.99	0.80	1.00	1.00
Exponential-peak	2.15	1.29	4.05	3.69	0.90	0.27	1.00	0.93

* *H*_0_ is true

**Figure 6:**
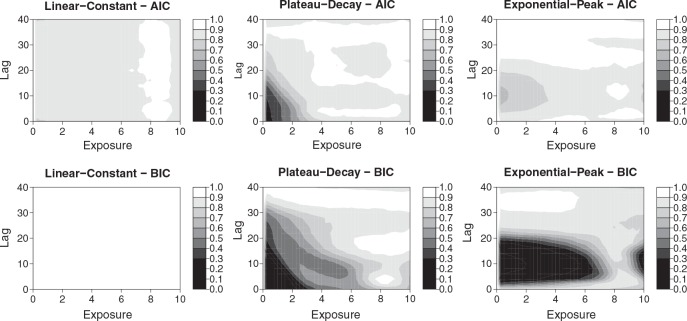
Empirical coverage across the risk surfaces for three scenarios of exposure–lag–response associations (linear-constant, plateau-decay, and exponential-peak, in each column). Results from *m* = 500 simulated data sets with *n*_*s*_ = 400 subjects.

The undercoverage of confidence intervals as shown in Table IV can be attributed to both lack of fit and a posteriori model selection. The latter, as discussed in Section 2.5, may generate undercoverage through the underestimation of the true sampling (co)variance. A comparison of the importance of the two sources can be provided by the assessment of undercoverage in the first scenario, where linear and constant functions are actually among the options of the selection procedure, and the underlying simulated association can be potentially recovered with no lack of fit. In this scenario, AIC-selected models affected by overfitting show a coverage of 91%, very close to the nominal value, as illustrated in Table IV. The under-coverage seems to be proportional to the bias, as confirmed by Figure 6, with a lower coverage corresponding to sections of the bidimensional space characterized by worse fit.

The simulated examples with *n*_*s*_ = 200 and *n*_*s*_ = 800 generate approximately 150 and 600 uncensored events, respectively. The versions of Tables IV–V and Figure 5 for these examples are reported in Tables S2–S5 and Figures S9–S10 of the supporting information. The comparison suggests that varying the sample size does not dramatically affect the performance of the AIC-based test, apart from the expected different power in identifying nonlinear and noncostant exposure–time–response associations. Consistently, AIC-based selection seems to perform well across the range of number of subjects included in the analysis, with a small bias and reasonable coverage. The results of this simulation study are consistent with previous findings on one-dimensional models for exposure–lag–response associations assuming a linear exposure–response relationship 18.

## 5. Discussion

In this contribution, I illustrate a statistical framework for modeling temporal dependencies with time-varying exposures, defined here as exposure–lag–response associations. The approach is based on the extension of distributed lag non-linear models, a modeling class previously proposed in time series analysis 23,24. The extended DLNM methodology brings together and extends previous methodological developments on the topic, as summarized in. Briefly, it provides a unified framework for different study designs and regression methods, and is applicable to time series, cross-sectional, case-control, survival, and longitudinal data. A major advantage is the possibility to describe the lag structure of either linear or nonlinear exposure–response relationships, through the choice of two functions that define the association along the dimensions of the predictor and lags, including most of the previous approaches as special cases. The example in illustrates how such flexibility is important for obtaining correct estimates of the association. Model specification easily accounts for previous knowledge on the association and incorporates assumptions on the phenomenon to be investigated, through the choice of specific functions, lag period and constraints. Interpretation of complex exposure–lag–response associations is aided by the definition of simple summary measures of effect and prediction, and by graphical representation. The modeling framework is defined through a neat and compact algebraic representation, including the derivation of measures of uncertainty such as standard errors and confidence intervals. Estimation is carried out with standard regression models, which do not require specialized optimization procedures, and may include terms for multiple exposure–lag–response dependencies, as shown for radon and smoking here. The parameterization, prediction and graphical representation are carried out with few general functions implemented in a freely available and documented software, as discussed in.

A key issue of the DLNM methodology is about selecting the appropriate model among different options for modeling the bidimensional exposure–lag–response relationship. The simulation study in indicates that AIC-based selection performs reasonably well over a range of 150–600 uncensored events, while the strong penalty of BIC induces the selection of models too simple to recover the underlying dependency. The overfitting characterizing AIC-selected models in scenarios of simple exposure–lag–response dependencies does not seriously affect its performance, a result in line with previous findings 18. However, AIC-selected models also suffer from bias and undercoverage of confidence intervals to some extent. Part of this seems to be related to the limited flexibility of the functions applied in the simulation study and may be described as a smoothing problem rather that an inherent limitation of the estimators. It should also be noted that the simulation study only evaluates a limited set of exposure–response and lag–response shapes, simulated under the assumption of independency. Different functions, such as cubic splines, and more complex exposure–lag–response surfaces will be assessed in future simulation studies. Also, an extension of DLNMs with penalized splines, characterized by higher flexibility can be explored as well, exploiting previous research on bivariate smoothing techniques 30,31.

A related problem is about the inferential procedures being conditional on a posteriori selection of the best-fitting model. Previous studies on unidimensional models have proposed a correction for the inflation of type I errors in tests on a constant effect along lags 17,27. However, this approach is not easily extended to the bidimensional setting of exposure–lag–response associations, and the definition of a hypothesis testing procedure for DLNMs is left to future developments. Although a posteriori selection may also be a source of undercoverage of confidence intervals, its impact seems to be limited if compared with that associated with lack of fit, at least in the simple scenarios investigated in the simulation study. Another limitation is the lack of a formal testing procedure on the hypothesis of independency. As suggested in Section 3.4, a graphical assessment of the proportionality of exposure–response and lag–response curves, such as those in Figure 3, can help investigating the issue. Further research is needed to provide more consistent inferential procedures in this setting.

The analysis of the temporal evolution of the risk associated with protracted time-varying exposures has straightforward applications in different research fields. For example, the DLNM methodology may be used to characterize the risk of chronic exposures to occupational or environmental factors, to differentiate the role of exposures sustained at different ages in life course studies, or to define the temporal frame of beneficial or adverse effects of drugs in clinical trials and pharmaco-epidemiology. The development of this methodology and software implementation provide a promising analytical tool for biomedical research.

## 6. Software and data

All the analyses presented in this paper were performed using the R software version 3.0.1 32. The DLNM modeling framework is fully implemented in the package dlnm 25, by using the expressly extended version 2.0.0. The permutational algorithm for simulating time-to-event data in the presence of time-varying exposures is implemented in the package PermAlgo 29, version 1.0. Both packages are available through R from its central repository. The data of the Colorado Plateau uranium miners cohort in the form of a comma-separated values file is included in the supporting information,^‡^ together with the R scripts for the analysis performed in the example and the simulation study of Sections 3–4, which are entirely reproducible. In particular, the script example.R provides a short illustration of the modeling framework. Versions of the scripts updated to future versions of the dlnm package will be available at http://www.ag-myresearch.com.
