# Supplementary material for: Modeling exposure–lag–response associations with distributed lag non-linear models
Source: Stat Med. 2013 Sep 12;33(5):881–99. doi: 10.1002/sim.5963 (PMC4098103; doi:10.1002/sim.5963)
Supplement: Supplementary file 1 [file sim0033-0881-SD1.pdf]

Research Article

Statistics in Medicine

Received XXXX

(www.interscience.wiley.com) DOI: 10.1002/sim.0000

Supplementary Web Appendix – "Modelling exposure-lag-response associations with distributed lag non-linear models"

Antonio Gasparrini[a*†]

This web appendix contains additional information on the DLNM methodology and on the analysis of the illustrative example and simulation study. Specifically, a summary of the main steps of the DLNM analysis is provided in Section A, while further information on the modelling approach is added in Section B. Data and software are described in Section C, while Section D provides additional results and information on the analysis of the example. Section E includes additional information on the simulation study. Updated information on the methodology and a version of the R code compatible with future version of the package dlnm can be found at my personal web-page www.ag-myresearch.com.

A. Steps of DLNM analysis

This section summarizes the main steps to be undertaken in the analysis of exposure-lag-response associations through DLNMs. The list provided below may help in replicating the analysis of the Colorado Plateau uranium miners cohort illustrated in Section 4 of the manuscript in other real-life applications. The analysis exploits the functions included in the dlnm package of R, as described in Section C.2. The R script `example.R`, included as supplementary material, provides a simple example which reproduces the main results.

The main steps are:

- *Data preparation*. For time-to-event or other longitudinal data, N subject-period observations need to be derived from the data on the n subjects, as detailed in Section 4.2 of the manuscript and in Section B.1 below. This step is not required for other types of data used in different study designs where each record relates to a single observation sampled in a specific time, such as case-control, time series or cross-sectional data.
- *Modelling choices*. The user must define the lag period (minimum and maximum lag) and the model candidates, namely the different options for the exposure-response function $f(x)$ and lag function $w(\ell)$ composing the lag-basis or cross-basis functions. In particular, this step identifies the type of function (linear, constant, B-splines, piecewise constant and threshold amongst others) and optional constraints. These choices are dictated by specific assumptions on the exposure-lag-associations. Multiple predictors can be modelled through lag-basis and/or cross-basis functions.
- *Generate exposure histories*. The matrix of exposure histories $\mathbf{Q}$ from Eq. 3 in the manuscript represents the exposure events related to each observation within the selected lag period. This can be reconstructed if not available, as in the example for the Colorado Plateau uranium miners cohort.
- *Compute the cross-basis matrix*. For each model, the lag-basis or cross-basis matrix $\mathbf{W}$ in Eq. 3 and 7 in the manuscript is computed with the function `crossbasis()` of the package dlnm. This function accepts as

[a] *Medical Statistics Department, London School of Hygiene and Tropical Medicine*
[*] *Correspondence to: Antonio Gasparrini, London School of Hygiene and Tropical Medicine, Keppel Street, London WC1E 7HT, UK.*
[†] *E-mail: antonio.gasparrini@lshtm.ac.uk*

Contract/grant sponsor: Medical Research Council (UK), grant G1002296

Copyright © 2011 John Wiley & Sons, Ltd.

arguments the matrix $\mathbf{Q}$, the selected lag period and the options for defining the functions $f(x)$ and $w(\ell)$, as selected above.

- *Fit the model.* The lag-basis or cross-basis matrix $\mathbf{W}$, containing the transformed variables, is included in the regression formulae of standard regression functions, such as `coxph()`, `clogit()`, `lm()`, `glm()`, `gee()`, `lme()` or `gam()`, for estimating the parameters $\boldsymbol{\eta}$.
- *Model comparison.* The fit of the models with different specifications of $f(x)$ and $w(\ell)$ can be compared through AIC and BIC, identifying the best-fitting model and assessing the evidence for a non-linear exposure-response and non-constant risk along lags.
- *Predict the exposure-lag-response surface.* Predictions from DLM and DLNMs are obtained from the function `crosspred()`, which accepts as arguments the cross-basis and model objects (or alternatively the selected coefficients and (co)variance matrix). This function returns the grid of risk contributions β_{x_p,ℓ_p} from Eq. 8–9, composing the risk surface for specific user-defined exposure and lag values, together with the overall cumulative effects.
- *Graphical representation.* The risk surface of the exposure-lag-response association, or specific exposure-response or lag-response curves, can be represented graphically with method functions `plot()`, `lines()` and `points()`, reproducing all the figures included in the manuscript. The comparison of different models can help the interpretation of the results.
- *Predict the risk for specific exposure histories.* The overall cumulative effects $\hat{\beta}_c$ in Eq. 10 for specific exposure histories can be also derived from `crosspred()`. The trend of overall cumulative risk over time can be similarly predicted, generating a matrix with time-varying exposure histories.

B. Additional information on modelling

B.1. Dealing with time-varying exposures in time-to-event data

As described in Section 3.2 of the manuscript, the analysis of the Colorado Plateau uranium miners cohort is performed using a Cox proportional hazard model. The specification of models for time-to-event data with continuous time-varying predictors, assumed by definition when modelling exposure-lag-response associations, requires specific analytical and computational strategies [1]. In particular, the analysis needs to account for the fact that the exposure of a given subject changes along time. A suggested method is to divide the follow-up period for each subject into short equally-spaced intervals, and to compute the exposure values for each of them. However this approach, previously used for exposure-lag-response analyses [2], may considerably expand the dataset in the case of extended follow-up periods. An alternative and less approximate approach takes advantage of the specific likelihood form of the Cox model, based on the sum of contributions of risk sets defined at each failure time [3]. The risk sets can be created by splitting the follow-up period of each subject at each failure time, generating subject/period observations. Each observation belongs to a single risk set, and its exit time corresponds to the exact moment the subject contributes to the risk set. In practice, for the likelihood to be defined, the computation of the time-varying exposure level can be limited to the exit time of each subject/period observation.

In addition, in exposure-lag-response relationships the relevant time-varying exposure is represented by the whole exposure history in a defined lag period. This is computed for each subject/period observation, given the exposure profile of each subject and the specific exit time. In the example included in the manuscript, the chosen time axis is age, and the exposure histories are reconstructed by following the method described in Section B.2. However, the split of the dataset generates 343,236 subject/period observations, introducing substantial computational problems. Actually, the function `coxph` still performs quite well with this number of observations, but the computation of the cross-basis, following Eq. 7 in the manuscript, is not manageable in terms of memory allocation. Although this step can be optimized regarding memory and computing time in future releases of the package dlnm, for this specific example I chose to reduce the volume of the data by sampling from the risk set.

This approach is thoroughly illustrated by Langholz and Goldstein [4]. In practice, each risk set is composed by one or few cases and usually a high number of subjects acting as controls. The number of subject/period observations can be reduced by randomly selecting a sub-sample of controls from each risk set. This approach resembles a matched case-control design nested within the cohort, where the inclusion of all the controls in each risk set produces the same likelihood of the standard cohort analysis. The number of sampled controls is proportional to the precision of the estimates compared to the full analysis. In the example illustrated in the manuscript, I selected all the controls in each risk set up to a maximum of 100, a number which guarantees a feasible computation and a negligible approximation. With this split dataset of 25,096 subject/period observations, the regression can be performed with both `coxph` or `clogit` functions, which return exactly the same results.

 Copyright © 2011 John Wiley & Sons, Ltd.

B.2. Reconstructing the exposure histories

For each subject, the histories of radon exposures and smoking are reported as cumulative measures in five-year age periods. First, the approximated exposure values at each age in years (from 0.5 to 99.5) are computed, accounting for sub-periods defined by age at start and end of mining/smoking. Then, the exposure history for lag 2–40 is retrieved by linking such age-specific values with age at exit of each of the 25,906 subject/period observations, generating the matrix $\mathbf{Q}$ in Eq. 2 of the manuscript. The approximation is to yearly exposures, where lag 0 refers to the cumulative exposure sustained in the last year, lag 1 between 1 and 2 years ago, and so on.

C. Data and software

C.1. The Colorado Plateau uranium miners cohort

The data for the Colorado Plateau uranium miners cohort includes information for 3,347 male subjects. Eligibility criteria were for the miner to have worked in the mines of the four-state Colorado Plateau area for at least one month, with at least one examination from public health service physicians between 1950 and 1960, and to have completed a questionnaire providing social and occupational information. The dataset used here refers to the follow-up at 31st December 1982, with 1,258 deaths (37.6%), including 258 lung cancer cases (7.7%) [5]. A more recent follow-up is also available [6]. Exposure data available in the dataset include cumulative measures of radon and smoking in five-year age intervals. At the time of writing, a detailed description of the cohort was available at hydra.usc.edu/timefactors/.

The data is available as a comma-separated values (.csv) file. The labels and descriptions of the 52 variables included in the dataset are summarized in Table S1.

C.2. The R package dlnm and the R code

The package dlnm contains functions to run the analyses illustrated in Section 3 of the manuscript. The analysis in the manuscript and the illustration below refer to version 1.6.6. Specifically, the function `crossbasis` generates the lag-basis and cross-basis matrices, explained by Eq. 1–7 in the manuscript, to be included in the regression function `coxph`. The function `crosspred` is used to predict the risk summaries in Eq. 8–10 for specific exposure values or exposure histories. The method functions `plot` and `lines` are called to graphically represent such risk summaries, producing the figures included in the manuscript.

The R scripts provided as supplementary material reproduce all the results illustrated in the manuscript, figures included. The R packages dlnm, xtable and PermAlgo, available in the R CRAN, need to be installed. The scripts are meant to be run consecutively. In particular, the script `example.R` provides a short and easy-to-follow illustration of the main steps of the analysis, if compared to the more convoluted programming used in the other scripts.

The package dlnm is under constant development, and the usage of existing functions may change, although portability of the existing code in future versions will be preserved whenever possible. An updated version of the code can be found at my personal web-page www.ag-myresearch.com.

D. Additional analyses

D.1. Specifying right-constrained models

As discussed in Section 2.4 of the manuscript, a right constraint on the lag-response curve can be specified by excluding specific variables of the B-spline basis for the space of ℓ. Figure S1 shows the four basis variables of a quadratic B-spline without intercept, defined in the support interval 0–40, with internal knots at 13.3 and 26.6. Basically, the constraint is applied by excluding the last two variables (green dash-dotted and blue long-dashed curves in Figure S1). This can be achieved by specifying a user-defined modification of the B-spline function, as shown in the R code included in the Supplementary Web Appendix.

An example is provided in Figure S2, where a left and right-constrained model is compared to the left-constrained Model 8 described in the manuscript. As expected, the two estimated exposure-lag-response associations are very similar, given that Model 8 already estimates a null risk at the end of the lag period. The double-constrained model shows a lower AIC of 2148.0.

Copyright © 2011 John Wiley & Sons, Ltd.

D.2. Analysis on a subset of subjects

The analysis is repeated using to the subset of subjects with a maximum yearly exposure to radon less than 300 WLM/year (81.6% of the total). The results are shown in Figure S3, showing a comparison of the estimated exposure-response curves and lag-response curves from Model 8 with the complete data, and those from Model 8 and Model 4 with the subset. The estimates for Model 8 are almost identical, indicating that the results from this more flexible option are not biased by few high exposure events. Although the fit of Model 4 and Model 8 in this subset of data is now more similar, the AIC still largely favours the latter, with values 1685.2 and 1659.2 respectively, suggesting that non-linearity is not limited to very high exposures.

D.3. Modelling the exposure-response with the log function

The analysis is also carried out by replacing the spline in Model 8 with the log function to model the exposure-response relationship, namely a DLNM with $f(x) = \log(x + 1)$. Again, this can be achieved by specifying a user-defined function to be used in the cross-basis definition, as shown in the R code included in the Supplementary Web Appendix. The AIC value of the new model and Model 8 are 2148.6 and 2153.2 respectively. However, Figure S4 indicates that the estimates are very similar.

D.4. Sensitivity analysis on knot location

An additional sensitivity analysis assesses the impact of knot location for the lag-exposure function $w(\ell)$ in Model 8. The best fitting model presented in the main text has a single knot at 13.3 lag. Models with alternative knot placements at 20, 26.6 and 13.3-26.6 lags (total *df* equal to 9, 9 and 12) display an higher AIC of 2154.7, 2157.9 and 2158.0 respectively. The comparison of the fit of alternative models is shown in Figure S5.

E. Additional information on the simulation study

E.1. Simulating the exposure profiles

A $n_s \times 100$ matrix of exposure profiles is produced for each of the three simulation settings, with number of subjects n_s equal to 200, 400 or 800. Each row of the matrix represents a series of exposure events x_t potentially experienced by a specific subject at times $t = 1, \ldots, 100$. The series is generated as 9–14 random exposure periods of length 1–10, with intensity between 0 and 10. The distribution of the exposure events across the n_s subjects and examples of exposure profiles from 3 random subjects are reported in Figure S6.

E.2. Simulating scenarios of exposure-lag-response associations

The scenarios of exposure-lag-response associations are defined through bi-dimensional functions $f_s(x) \cdot w_s(\ell)$, generating risk contributions for each value of exposure x and lag ℓ. Three different shapes are generated for $f_s(x)$ and $w_s(\ell)$ through simple mathematical functions. Specifically, the exposure-response function $f_s(x)$, defined for $x \in [0, 10]$, is specified as *linear*, *plateau* and *exponential*, with:

$$\begin{aligned} \text{Linear} &= x/18 \,, \\ \text{Plateau} &= \Big(1 - \frac{1 + x/1.5}{(1 + x/1.5)^2}\Big)/1.9 \,, \\ \text{Exponential} &= \frac{e^{x/3.5} - 1}{e^{10/3.5}}/1.1 \,. \end{aligned} \quad (1)$$

The lag function $w_s(\ell)$, defined for $\ell \in [0, 40]$, is specified as *constant*, *decay* and *peak*, with:

$$\begin{aligned} \text{Constant} &= 0.20 \,, \\ \text{Decay} &= e^{-\ell/9} \cdot 0.95 \,, \\ \text{Peak} &= \frac{1}{7 \cdot 2\pi} \cdot e^{-\frac{1}{2}(\frac{x-10}{7})^2} \cdot 8.5 \,. \end{aligned} \quad (2)$$

These shapes are illustrated in Figure S7. Nine simulated scenarios of exposure-lag-response associations are then specified with $f_s(x) \cdot w_s(\ell)$ including all possible combinations of $f_s(x)$ and $w_s(\ell)$ as above. The nine bi-dimensional dependencies are illustrated in Figure S8. These associations generate different distributions of overall cumulative risk $\beta_{c,i}$ for random exposure histories, with a median of HR ranging approximately from 2.5 to 4.5.

 Copyright © 2011 John Wiley & Sons, Ltd.

E.3. *Sampling time-to-event data*

Time-to-event data are simulated in each of the $m = 500$ data sets as exit time and type of event (censored or uncensored) for the n_s subjects, using a permutational algorithm developed for simulating time-to-event data in the presence of time-varying exposures [7]. Event times are generated conditional on the cumulative contribution of the exposure history at each time t, computed as $s_s(x,t) = \sum_\ell f_s(x_{t-\ell}) \cdot w_s(\ell)$ over lag $\ell = 0, \ldots, 40$, with alternative functions f_s and $w(\ell)$ selected in different scenarios.

The algorithm is implemented in the R package PermAlgo, and involves three steps. First, the individual time-varying cumulative contribution is computed from $s_s(x,t)$ for each time $t = 1, \ldots, 100$ for each of the n_s subjects, given the associated time-varying exposure history. In the second step, event and censoring times are randomly sampled from uniform distributions with limits 0–100 and 0–200, respectively, thus producing approximately 25% of censored observations. Finally, event and censoring times are matched with the time-varying cumulative contributions, proceeding from the earliest to the latest times, to define the related risk sets. In censoring times, a censored subject is randomly selected with equal probability across the risk sets. In event times, the case is randomly sampled with probabilities conditional on the time-varying cumulative contributions, simply assigning to this covariate a coefficient equal to 1. Additional details are provided elsewhere [7].

E.4. *Additional simulation results*

Results for the simulations study reported in tables and graphs in the manuscript refer to the data sets with $n_s = 400$ subjects. The versions of Tables 4 and 5 in the manuscript with $n_s = 200$ and $n_s = 800$ are included here in Tables S2–S5, while versions of Figure 5 in the manuscript are included as Figures S9–S10.

References

1. Breslow NL, Day NE. *Statistical Methods in Cancer Research*, vol. II: The desing and analysis of cohort studies, chap. 5: Fitting models to continuous data. International Agency for Reasearch on Cancer (IARC): Lyon, 1987; 178–231.
2. Sylvestre MP, Abrahamowicz M. Flexible modeling of the cumulative effects of time-dependent exposures on the hazard. *Statistics in Medicine* 2009; **28**(27):3437–3453.
3. Cox DR. Regression models and life-tables. *Journal of the Royal Statistical Society: Series B* 1972; **34**(2):187–220.
4. Langholz B, Goldstein L. Risk set sampling in epidemiologic cohort studies. *Statistical Science* 1996; **11**(1):35–53.
5. Hornung RW, Meinhardt TJ. Quantitative risk assessment of lung cancer in US uranium miners. *Health Physics* 1987; **52**(4):417–430.
6. Roscoe RJ. An update of mortality from all causes among white uranium miners from the Colorado Plateau Study Group. *American Journal of Industrial Medicine* 1997; **31**(2):211–222.
7. Sylvestre MP, Abrahamowicz M. Comparison of algorithms to generate event times conditional on time-dependent covariates. *Statistics in Medicine* 2008; **27**(14):2618–2634.

Copyright © 2011 John Wiley & Sons, Ltd.

Table S1. Variables included in the dataset of the Colorado Plateau uranium miners cohort. Exposure to radon is measured in working level months (WLM), while smoking is reported as packs of cigarettes/100.

Variable	Name	Description
1	record	Sequential record number
2	ind	1=lung cancer death, 0=otherwise
3	agest	Age at entry to study (in yrs)
4	ageexit	Age at exit from study (in yrs)
5	bmon	Birth month
6	byr	Birth year (3 digits, e.g. 912=1912)
7	dmon	Death month
8	dyr	Death year (3 digits)
9	status	0=alive, 1=dead, 9=missing
10	totrdn	Total cumulative radon
11	rdnstar	Age at start of mining (in yrs)
12	rendage	Age at end of mining (in yrs)
13	priorex	Prior exposure
14	totsmk	Total cumulative smoking (100s of packs)
15	smkstar	Age started smoking (in yrs)
16	sendage	Age last known to smoke (in yrs)
17	rexp5	Total radon exposure during ages 0-4
⋮	⋮	⋮
34	rexp90	Total radon exposure during ages 85-89
35	sexp5	Total smoking exposure during ages 0-4
⋮	⋮	⋮
52	sexp90	Total smoking exposure during ages 85-89

Table S2. Version of Table 4 in the manuscript with $n_s = 200$ subjects.

	$\exp(\text{Bias}) - 1$		Coverage		$\exp(\text{RMSE}) - 1$	
$f(x) \cdot w(\ell)$	AIC	BIC	AIC	BIC	AIC	BIC
Linear-Constant	0.04	0.04	0.91	0.94	0.15	0.09
Linear-Decay	0.03	0.03	0.92	0.93	0.17	0.12
Linear-Peak	0.03	0.09	0.95	0.85	0.15	0.18
Plateau-Constant	0.03	0.11	0.85	0.78	0.14	0.13
Plateau-Decay	0.02	0.16	0.87	0.75	0.19	0.22
Plateau-Peak	0.05	0.11	0.82	0.70	0.22	0.24
Exponential-Constant	0.04	0.11	0.87	0.82	0.18	0.17
Exponential-Decay	0.04	0.12	0.92	0.82	0.22	0.25
Exponential-Peak	0.08	0.21	0.89	0.77	0.26	0.29

Copyright © 2011 John Wiley & Sons, Ltd.

Table S3. Version of Table 4 in the manuscript with $n_s = 800$ subjects.

	$\exp(\text{Bias}) - 1$		Coverage		$\exp(\text{RMSE}) - 1$	
$f(x) \cdot w(\ell)$	AIC	BIC	AIC	BIC	AIC	BIC
Linear-Constant	0.02	0.02	0.93	0.96	0.03	0.02
Linear-Decay	0.00	0.00	0.92	0.93	0.04	0.03
Linear-Peak	0.01	0.01	0.93	0.93	0.03	0.03
Plateau-Constant	0.04	0.10	0.89	0.74	0.03	0.06
Plateau-Decay	0.04	0.10	0.89	0.82	0.05	0.07
Plateau-Peak	0.05	0.18	0.88	0.61	0.06	0.12
Exponential-Constant	0.02	0.01	0.93	0.81	0.04	0.05
Exponential-Decay	0.04	0.06	0.91	0.90	0.07	0.06
Exponential-Peak	0.04	0.03	0.92	0.78	0.07	0.09

Table S4. Version of Table 5 in the manuscript with $n_s = 200$ subjects.

	Average *df*				Empirical rejection rate			
	$f(x)$		$w(\ell)$		$H_0 : f(x) = x$		$H_0 : w(\ell) = c$	
$f(x) \cdot w(\ell)$	AIC	BIC	AIC	BIC	AIC	BIC	AIC	BIC
Linear-Constant	1.53	1.06	1.62	1.01	0.30*	0.04*	0.24*	0.00*
Linear-Decay	1.29	1.01	3.51	3.10	0.19*	0.01*	1.00	0.99
Linear-Peak	1.23	1.02	3.81	2.83	0.16*	0.02*	0.97	0.70
Plateau-Constant	1.94	1.32	1.52	1.03	0.62	0.28	0.20*	0.01*
Plateau-Decay	2.24	1.28	3.39	3.06	0.85	0.27	1.00	0.99
Plateau-Peak	1.85	1.20	3.76	2.73	0.62	0.17	0.97	0.68
Exponential-Constant	2.00	1.36	1.53	1.01	0.68	0.32	0.20*	0.00*
Exponential-Decay	2.21	1.43	3.43	3.16	0.90	0.42	1.00	1.00
Exponential-Peak	1.89	1.28	3.81	2.94	0.69	0.25	0.97	0.72

* H_0 is true

Table S5. Version of Table 5 in the manuscript with $n_s = 800$ subjects.

	Average *df*				Empirical rejection rate			
	$f(x)$		$w(\ell)$		$H_0 : f(x) = x$		$H_0 : w(\ell) = c$	
$f(x) \cdot w(\ell)$	AIC	BIC	AIC	BIC	AIC	BIC	AIC	BIC
Linear-Constant	1.42	1.01	1.68	1.01	0.26*	0.01*	0.25*	0.00*
Linear-Decay	1.26	1.00	3.74	3.25	0.18*	0.00*	1.00	1.00
Linear-Peak	1.17	1.00	4.08	3.96	0.12*	0.00*	1.00	1.00
Plateau-Constant	2.56	1.79	1.34	1.00	0.97	0.69	0.13*	0.00*
Plateau-Decay	2.75	1.94	3.60	3.07	1.00	0.90	1.00	1.00
Plateau-Peak	2.47	1.40	4.08	3.95	0.98	0.40	1.00	1.00
Exponential-Constant	2.38	1.70	1.34	1.00	0.95	0.68	0.13*	0.00*
Exponential-Decay	2.41	2.00	3.71	3.13	1.00	0.99	1.00	1.00
Exponential-Peak	2.30	1.53	4.08	3.96	1.00	0.53	1.00	1.00

* H_0 is true

Copyright © 2011 John Wiley & Sons, Ltd.

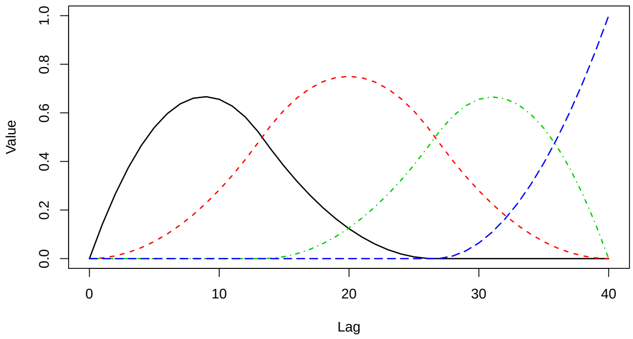

Figure S1. Values of the four basis variables for a quadratic B-spline function with internal knots at 13.3 and 26.6 (vertical lines), without intercept.

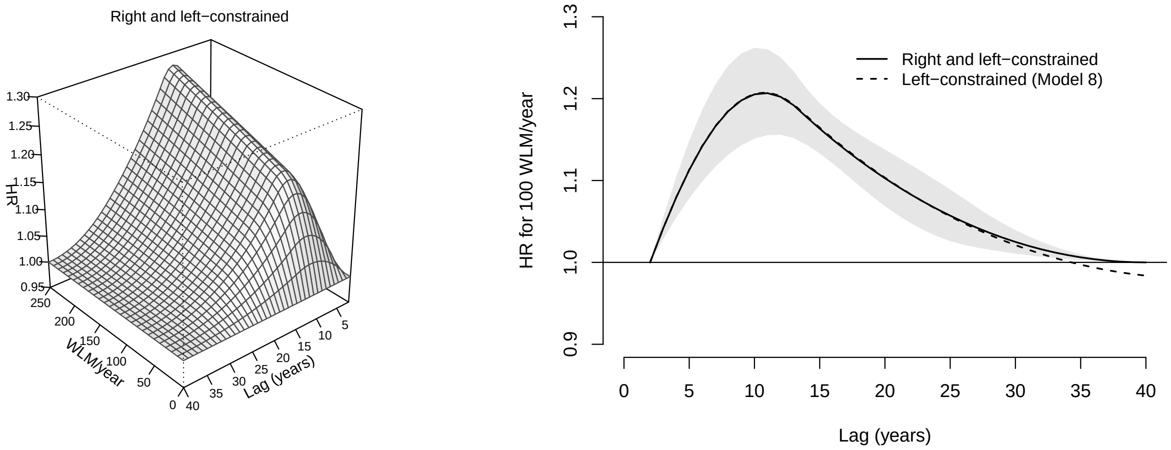

Figure S2. Hazard ratio (HR) of lung cancer mortality associated with radon exposure in the range 0–250 WLM/year and lag period 0–40 years. The figure shows 3-D graph of the exposure-lag-response association on a grid of exposure × lag values (left) and the lag-response curve for radon exposure of 100 WLM/year, for a left and right-constrained model, including a comparison with Model 8 in the manuscript. Data from the Colorado Plateau uranium miners cohort.

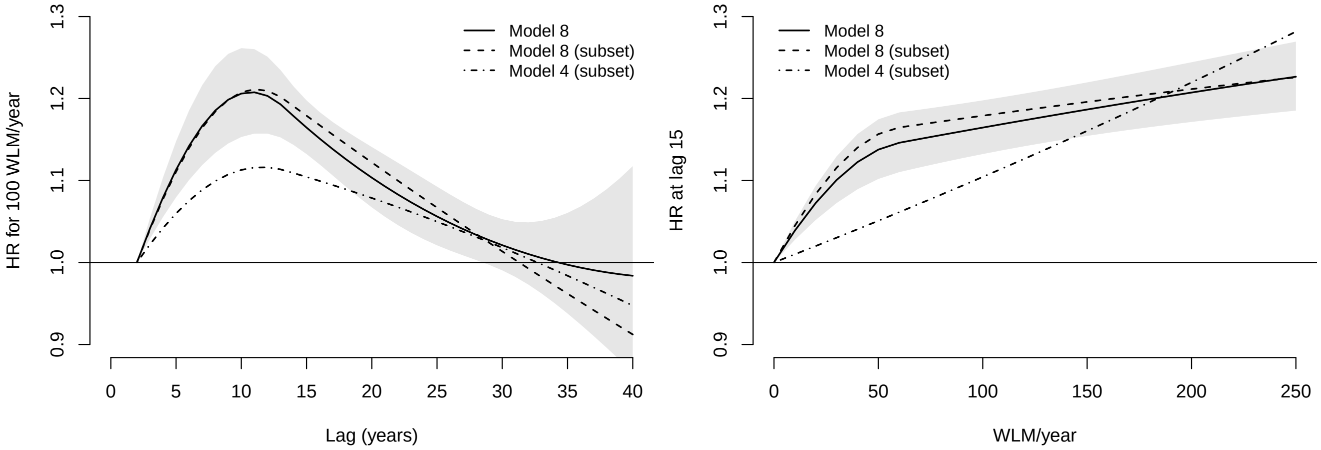

Figure S3. Hazard ratio (HR) of lung cancer mortality associated with radon exposure in the range 0–250 WLM/year and lag period 0–40 years. The figure shows lag-response curves for radon exposure of 100 WLM/year (left) and exposure-response curves (right) for Model 8 with complete data and for Models 8 and 4 in the subset of subjects with a maximum yearly exposure to radon less than 300 WLM/year. Data from the Colorado Plateau uranium miners cohort.

Copyright © 2011 John Wiley & Sons, Ltd.

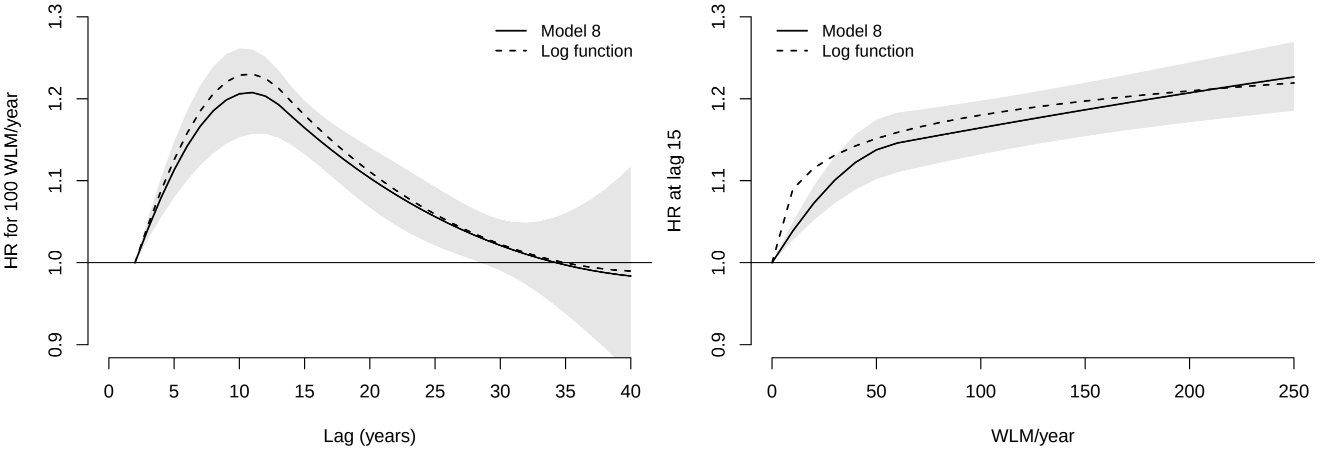

Figure S4. Hazard ratio (HR) of lung cancer mortality associated with radon exposure in the range 0–250 WLM/year and lag period 0–40 years. The figure shows lag-response curves for radon exposure of 100 WLM/year (left) and exposure-response curves (right) for Model 8 with untransformed exposure and for Models 8 and 4 with log-transformed exposure. Data from the Colorado Plateau uranium miners cohort.

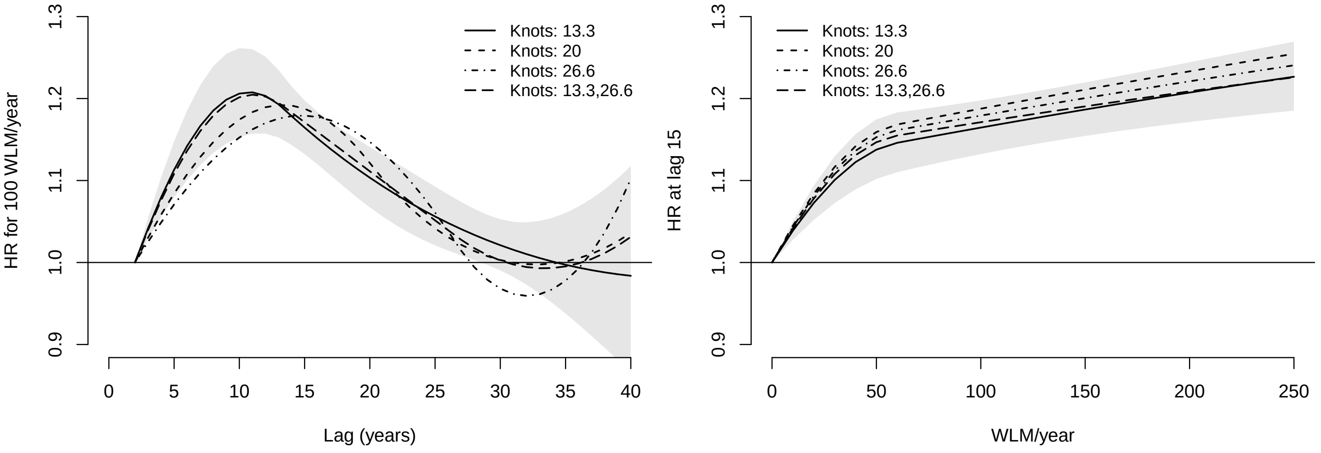

Figure S5. Hazard ratio (HR) of lung cancer mortality associated with radon exposure in the range 0–250 WLM/year and lag period 0–40 years. The figure shows lag-response curves for radon exposure of 100 WLM/year (left) and exposure-response curves (right) for variations of Model 8 with alternative knot locations for the lag-response function. Data from the Colorado Plateau uranium miners cohort.

Copyright © 2011 John Wiley & Sons, Ltd.

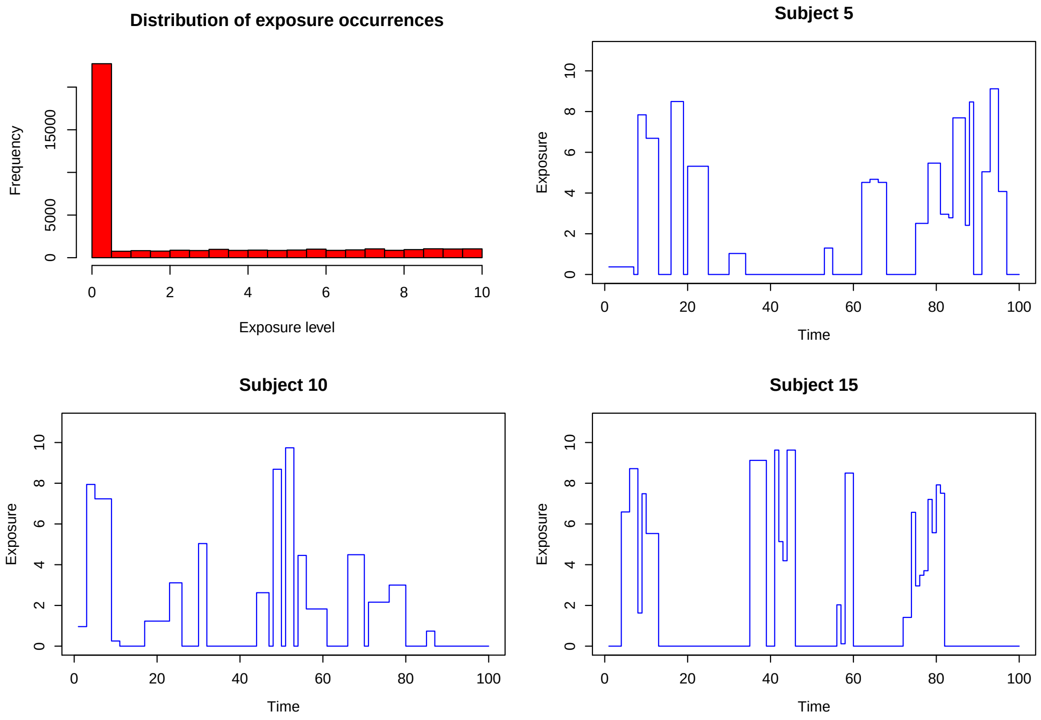

Figure S6. Distribution of the exposure events across the n_s subjects (top left panel), and examples of exposure profiles from 3 random subjects (the other three panels).

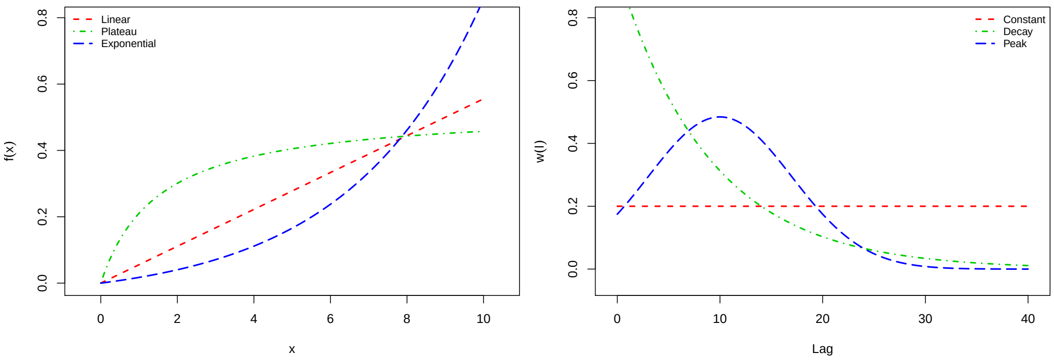

Figure S7. Shapes of the exposure-response and lag functions used for simulating the nine exposure-lag-response scenarios.

Copyright © 2011 John Wiley & Sons, Ltd.

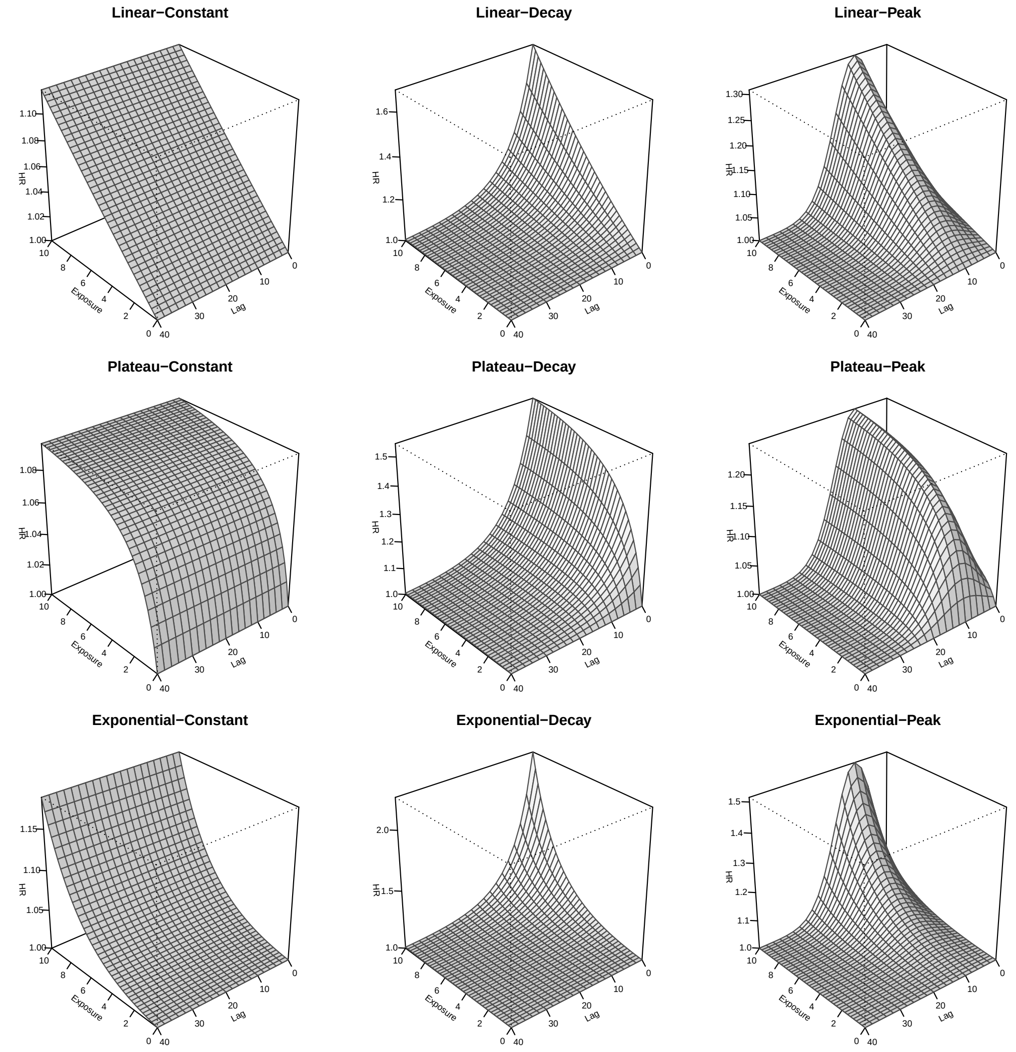

Figure S8. Bi-dimensional exposure-lag-response associations used in the nine simulated scenarios

Copyright © 2011 John Wiley & Sons, Ltd.

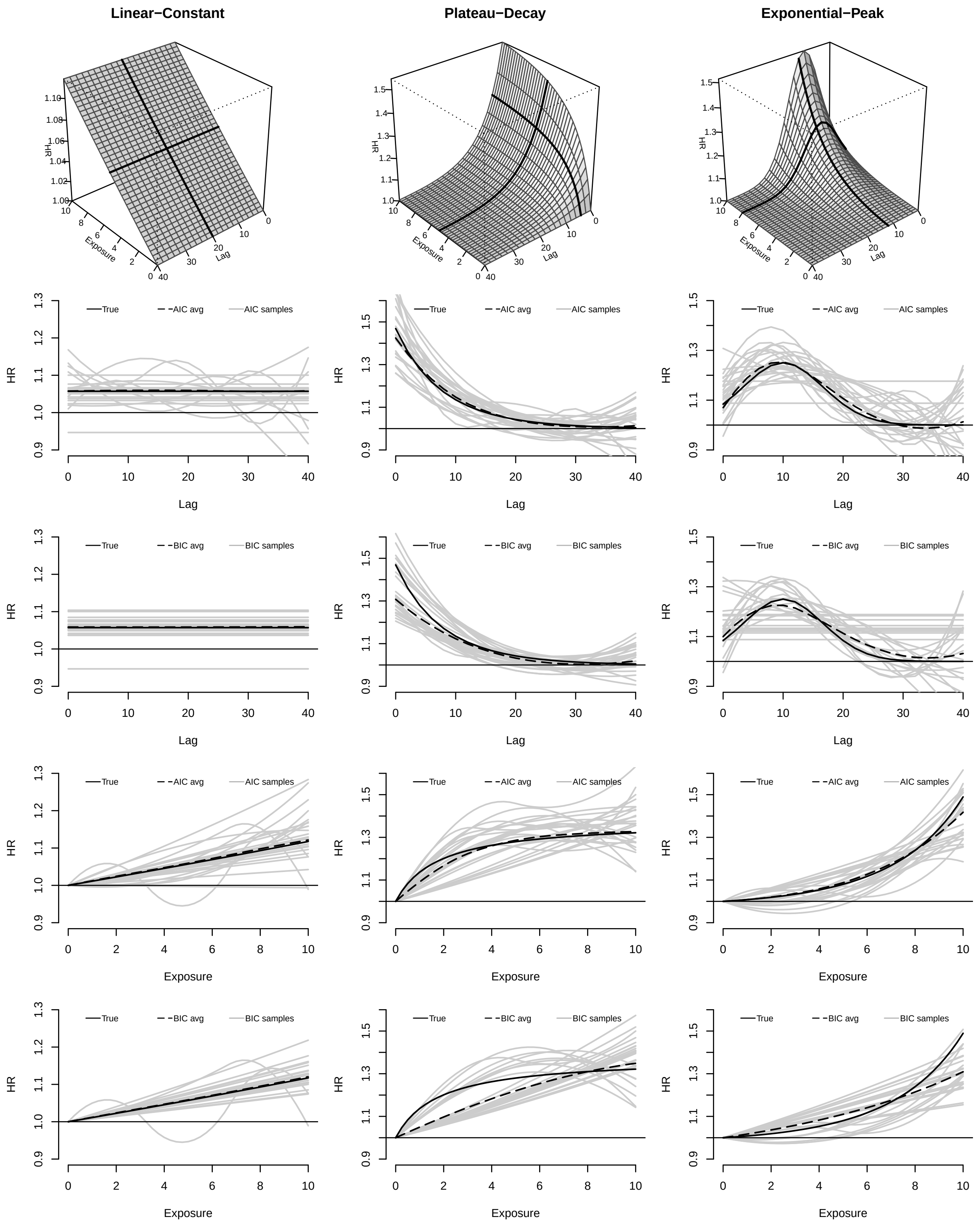

Figure S9. Version of Figure 5 in the manuscript for $n_s = 200$.

Copyright © 2011 John Wiley & Sons, Ltd.

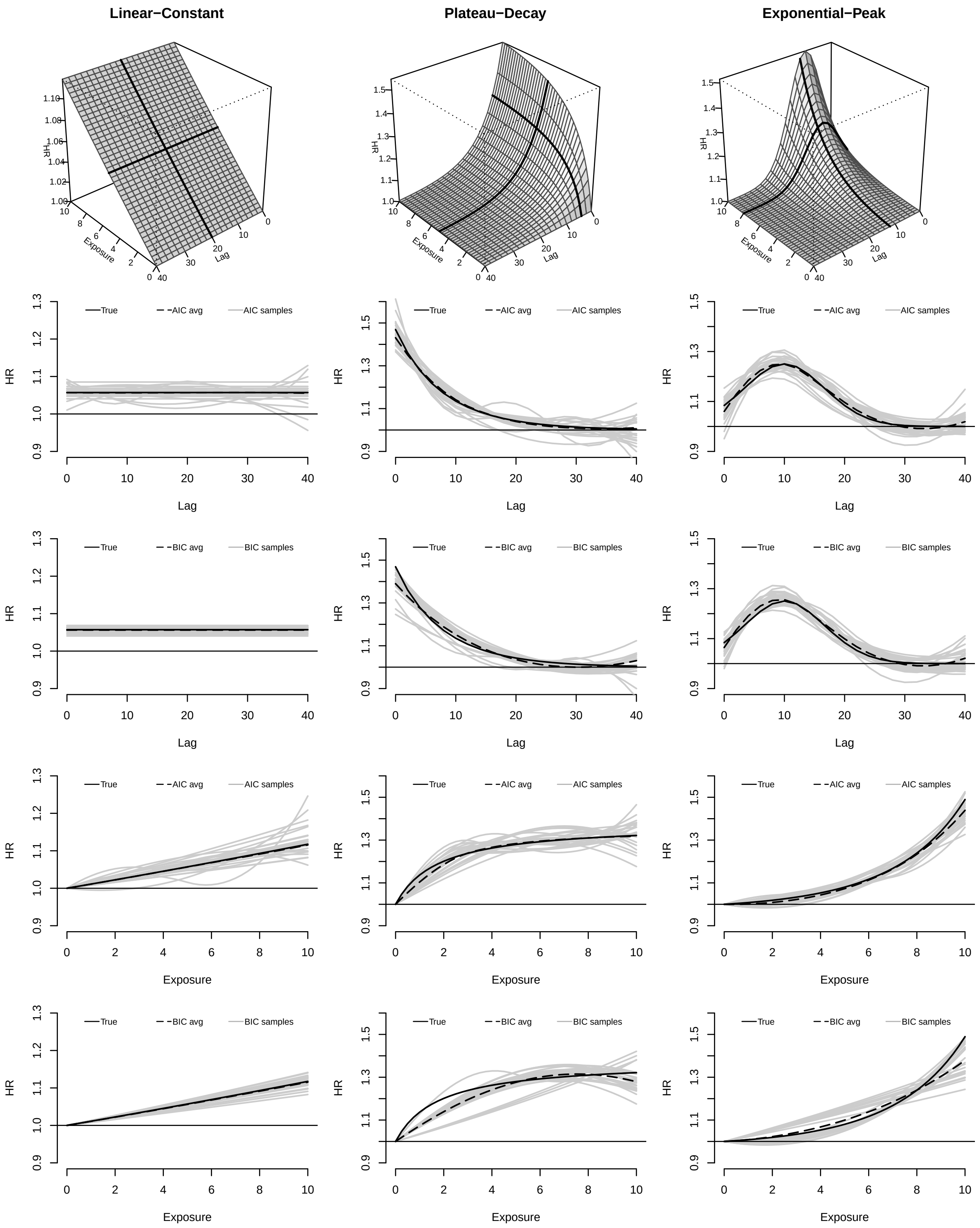

Figure S10. Version of Figure 5 in the manuscript for $n_s = 800$.

Copyright © 2011 John Wiley & Sons, Ltd.
